# Supplementary material for: Prevalence of Orthodontic Malocclusions in Healthy Children and Adolescents: A Systematic Review
Source: Int J Environ Res Public Health. 2022 Jun 17;19(12):7446. doi: 10.3390/ijerph19127446 (PMC9223594; doi:10.3390/ijerph19127446)
Supplement: Supplementary file 1 [file ijerph-19-07446-s001.zip › ijerph-1730536-supplementary.pdf]

Table of contents: additional files

Additional table S1: Excluded studies and reason for exclusion. 2

Additional table S2: Prevalence of Angle classification and deciduous molar occlusion. 4

Additional table S3: Prevalence of different transversal malocclusions and anterior crossbite. 7

Additional table S4: Prevalence of tooth anomalies. 10

Additional table S5: Prevalence of space anomalies 13

Additional table S6: Prevalence of Angle classification and deciduous molar malocclusion according to geographical location. 16

Additional table S7: Prevalence of different transversal malocclusions and anterior crossbite according to geographical location. 20

Additional table S8: Prevalence of tooth anomalies according to geographical location. 24

Additional table S9: Prevalence of space anomalies according to geographical location. 28

Additional file S10: protocol systematic review 32

Additional file S11: search strings 42

Additional file S12: prisma 2020 checklist 48

Additional table S1: Excluded studies and reason for exclusion.

Author Year	Reason for exclusion
Aldrees, A. 2012, Alvarado, K. 2017, Amini, F. 2013, Anistoroaei, D. 2019, Arai, K. 2019, Arandi, N. 2018, Baca-Garcia, A. 2004, Bassiouny, D. 2016, Bereket, C. 2011, Borzabadi-Farahani, A. 2011, Bourzgui, F. 2012, Bozga, A. 2014, Brunnelle, J. 1996, Celikoglu M. 2010, Celikoglu, M. 2010, Celikoglu, M. 2010, Celikoglu, M. 2010, Celikoglu, M. 2012, Chan, G. 2019, Chung, C. 2008, Colak, H. 2013, Fekonja, A. 2015, Fernandez, C. 2018, Fujita, Y.2009, Gabris, K. 2006, Garib, D. 2010, Giordano, A. 2019, Gkantidis, N 2017, Gokkaya, B. 2016, Gungor, K. 2016, Higashihori, N. 2018, Hirakata,C. 2016, Kamiloglu, B. 2014, Kamiloglu, B. 2014, Kazanci, F. 2011, Khandelwal, P. 2018, Krooks, L. 2016, la Monaca, G. 2019, Lai, C. 2013, Mazinis, E. 2012, Mossaz, J. 2014, Nunes Neto, T. 2014, Onyeaso, C. 2002, Ota, K. 2015, Otuyemi, O. 1999, Rashid, H. 2016, Reboucas, A 2017, Rizkallah, J. 2013, Saeed, T. 2014, Sanu, O. 2012, Sharma, G. 2016, Silveira, M. 2016, Simsek-Kaya, G. 2011, Souza-Silva, B. 2018, Thongudomporn, U. 1998, Topkara, A. 2011, Topkara, A. 2012, Trakiniene, G. 2013, Umweini, A. 2002, Umweni, A. 1997, Uslu, O. 2009, Vahid-Dastjerdi, E. 2011, van Wyk, P. 2004, Willems, G. 2001, Yan-Vergnes, W. 2013, Zhang, J. 2015	Age > 18 years
Abe, R. 2010, Abu Alhaija, E. 2004, Abu Alhaija, E. 2019, Aishwarya Reddy, A. 2018, Alam, M. 2014, Al-Nimri, K. 2011, Alquerban, A. 2015, Anosike, A. 2010, Barone, A. 1997, Bauman, J. 2018, Beane, R. 2003 Bell, R. 2014, Bellot-Arcis, C. 2013, Bissar, A. 2007, Bresnahan, B. 2010, Buyuk, S. 2017, Cabrita, J. 2017, Camargo, I. 2016, Caruso, S. 2019, Celikoglu, M. 2012, Celikoglu, M. 2011, Cirulli, N. 2019, Citak, M. 2016, Danielsen, J. 2015, de Amorim, C. 2018, De Oliveira Gomes, C. 2008, De Paula Junior, D. 2018, Dhamo, B. 2019, Dhamo, B. 2016, dos Santos Jacintho Lima, A. 2017, dos Santos, S. 2009, e Silva, L. 2016, Ephraim, R. 2015, Evans, R. 1988, Facal-Garcia, M. 2002, Fernandez, C. 2018, Filius, M. 2019, Finkelstein, T. 2015, Germa, A. 2016, Germa, A. 2010, Gomez de Diego, R. 2017, Grippaudo, C. 2013, Grippaudo, C. 2014, Grippaudo, C. 2019, Gunduz, K. 2015, Hintze, H. 1990, Kaieda,A. 2019, Kenrad, J. 2013 Koruyucu, M. 2014, Lagana, G. 2018, Lara, T. 2013, Larsson, E. 2001 Lempesi, E. 2014, Llompart, G. 2010, Lux, C. 2008, Mani, S. 2014, Marquezan, M. 2011, Mishra, A. 2017, Mucedero, M. 2013, Palma, C. 2003, Peres, K. 2013, Popovic, N. 2014, Rafflenbeul, F. 2019, Ramachandra Prabhakar, R. 2014, Salbach, A. 2012, Schatz, J. 2013, Shelton, A. 2008, Stahl, F. 2005, Suliano, A. 2007, Tuerlings, V. 2004, Tunç, E. 2011, Urzal, V. 2013, Wheeler, T. 1994, Wyne, A. 2001, Yan, B. 2013	Subject too specific
Fekonja, A. 2017, Markovic, E. 2020, Nik Husseini, N. 1996, Sayin, M. 2004	No age range
Beliaev, V. 2015, Fu, M. 2002, Gabris, K. 2001, Gabris, K. 2001, Kalina, A. 2015, Kepes, D. 2013, Kitai, N. 1990, Lee, J. 2011, Legovic, M. 2005, Legovich, M. 2001, Sobouti, F. 2016, Viskovic, R. 1988, Viskovic, R. 1990, Xu, T. 2019	Language
Andreeva, R. 2016, Ang, G. 2014, Ansai, T. 1993, Bässler-Zeltmann, S. 1998, Baubiniene, D. 2009, Candido Brizon, V. 2013, Damle, D. 2014, Dimberg, L. 2016, Feldens, C. 2015, Ferraz Mello, S. 2014, Foster Page, L. 2005, Fu, P. 2012, Ghabrial, E. 1998, Hammad, S. 2011, Hong, M. 2020, Ingelssondahlstrom, M. 1994, Jayashri, P. 2019, Jenny, J. 1991, Karim, A. 2015, Kim, Y. 2012, Korhonen, M. 2003, Liepa, A. 2003, Liu, J. 2013, Manzanera,D. 2009, Marques, C. 2007, Melink, S. 2010, Monteiro, A. 2017, Mugonzibwa, E. 2004, Mugonzibwa, E. 2008, Nalcaci, R. 2012, Nobile, C. 2007, Ovsenik, M. 2007, Ovsenik, M. 2007, Ovsenik, M. 2007, Silvestrini Biavati, A. 2011, Singh, S. 2016, Souames, M. 2006, Sultana, S. 2019, Tetradis, S. 1999, Tolessa, M. 2020, Ucuncu, N. 2001, Ugur, T. 1998, Vishnoi, P. 2017, Yang, J. 2019, Yavuz, M. 2007	No extra orthodontic features
Macias Gil, R. 2014	No sample size
al Nimri, K. 2000, Al-Azemi, R. 2010, Almerich-Silla J. 2014, Borzabadi-Farahani, A. 2011, Borzabadi-Farahani, A. 2009, Borzabadi-Farahani, A. 2009, Cortes, F. 2007, Finkelstein, T. 2019, Gomes, R. 2010, Josefsson, E. 2007, Keski-Nisula, K. 2003, Migale, D. 2009, Ommar, S. 2009, Peres, K. 2015, Prasad, M. 2016, Ringqvist, M. 1969, Sidlauskas, A. 2009, Yordanova, G. 2015	Part of sample treated patients
Al-Ani, A. 2017, Ali, B. 2016, Bjerklin, K. 1994, Caprioglio, D. 1990, Cardoso, M. 2015, D'Onofrio, L. 2019, Dallel, I. 2018, Danaei, S. 2006, Machuca, C. 1998, Nadejda, B. 2021, Oluranti, . 2009, Rehki, A. 2018, Tarallo, D. 1998 Thornton, J. 1996	Type article
Dimberg, L. 2013, Lagana, G. 2013	Duplicate publication
Abu Affan, A. 1990, Barata, A. 2018, Bhateja, N. 2014, Borutta, A. 1992, Burhan, A. 2016, Dominguez Fleites, L. 1982, Hannuksela, A. 1977, Hlongwa, P. 2005, Hussain, S. 2010, Ingervall,B. 1972, Jarvinen, S. 1977, Kawala, B. 2009, Legovic, M. 1990, Martins, M. 2009, Omaña-Vidal, E. 1986, Onyeaso, C. 2004, Payette, M. 1989, Pereira, W. 2020, Raftowicz-Wojcik, K. 2010, Raftu, G. 2018, Rolling, S. 1978, Sahoo, N. 2019, Shamaa Anjum, A. 2020, Schlegel, K. 1986, Sincar, D. 2020,	Not retrievable

<u>Legend additional table S1</u>: This table gives the reason of exclusion for each article (Author and year of publication) . The reason of exclusion are the following: Age > 18 year (even if only part of the sample is older than 18 years), subject too specific (not focusing on prevalence of malocclusion and different orthodontic features), No age range (if no age range or mean age are provided), Language (others than English, French, Dutch, German, Spanish and Portuguese), No sample size (if not provided), Part of sample treated patients (only included if study was performed on pre)treatment records), Type article (others than epidemiological studies,randomized controlled trials, clinical trials and comparative studies), Duplicate publication, not retrievable.

Additional table S2: Prevalence of Angle classification and deciduous molar occlusion.

Author,Year of publication	Population			Molar occlusion								
	Continent	Number of subjects	Age in Y	Class I	Class I mal-occlusion	Class II	Class II, 1	Class II,2	Class III	FTP	DS	MS
**Abu Alhaija, 2005** (12)	Asia	1003	13-15		55,3%	17,5%			1,5%			
**Abumelha, 2018** (13)	Asia	526	6-12		65,0%		12,9%	1,7%	8,4%			
**Alajlan, 2019** (14)	Asia	520	7-12		7,4%	32,3%			8,3%			
**al-Emran, 1990** (17)	Asia	500	13.5-14.5			16,4%			3,0%			
**Arabiun, 2014** (21)	Asia	1338	14-18	74,7%	12,7%	9,9%	4,8%	5,2%	1,0%			
**Baral, 2014** (25)	Asia	506	3-5							33,5%	8,5%	40,3%
**Behbehani, 2005** (28)	Asia	1299	13-14		57,8%	31,2%			11,0%			
**Bhayya, 2011** (31)	Asia	1000	4 – 6							52,5%	8,4%	35,9%
**Bilgic, 2015** (32)	Asia	2329	12-16	10,1%	34,9%		40,0%	4,7%	10,3%			
**Bourzgui, 2012** (33)	Africa	1000	8-12	61,4%		24,0%			10,0%			
**Campos-Arias, 2013** (35)	America	88	7.0*			10,2%			11,3%			
**Chauhan, 2013** (37)	Asia	1188	9-12	90,9%								
**Coetzee, 2000** (39)	Africa	214	3-8							18,2%	1,9%	65,9%
**Cosma, 2017** (40)	Europe	172	3-6							58,0%	5,0%	36,0%
**Dacosta, 1999** (41)	Africa	1028	11-18	12,3%	84,0%		1,7%		2,1%			
**de Almeida, 2008** (43)	America	344	3.94*							84,3%	9,7%	6,0%
**Dimberg, 2015** (46)	Europe	277	3 & 7 & 11.5		74,7%	20,9%	5,7%	1,8%	4,4%			
**Ferro, 2016** (51)	Europe	380	14	86,3%		19,5%			3,9%			
**Gàbris, 2006** (54)	Europe	483	16-18	52,8%								
**Gois, 2012** (55)	America	212	8-11	8,5%	54,2%	28,3%			9,0%			
**Grabowski, 2007** (56)	Europe	3041	4.5* & 8.3*		44,7%	28,6%			2,6%			
**Gudipaneni, 2018** (58)	Asia	500	7-12	52,8%		31,8%			15,4%			
**Howell, 1993** (63)	Oceania	154	13-17	65,0%			15,0%	12,0%	7,0%			
**Ingervall, 1975** (64)	Europe	200	8-16			14,5%			0,5%			
**Jerez, 2014** (66)	America	120	3-6	1,7%						44,1%	2,9%	44,1%
**Kabue, 1995** (69)	Africa	221	3-6							53,0%	0,0%	44,0%

**Kalbassi, 2019** (70)	Asia	1208	7-15		46,0%	36,3%			4,4%			
**Kasparviciene, 2014** (71)	Europe	709	5-7	10,1%		10,6%			0,8%	20,9%	24,9%	50,2%
**Komazaki, 2012** (74)	Asia	963	12-15	51,3%		12,3%	18,5%	7,5%	10,5%			
**Lagana, 2013** (75)	Europe	2617	7-15	40,4%		29,2%			3,2%			
**Lux, 2009** (78)	Europe	494	8.6- 9.6			10,2%			3,3%			
**Madiraju, 2021** (79)	Saudi Arabia	282	8-9	75,2%		23,4%			1,4%			
**Martins, 2009** (80)	America	1612	10-12	25,8%	47,7%		17,0%	5,3%	4,2%			
**Mohamed, 2014** (84)	Asia	106	8-10	39,6%		49,1%			11,3%			
**Mtaya, 2009** (85)	Africa	1601	12-14	93,6%		4,4%			2,0%			
**Mtaya, 2017** (86)	Africa	253	3-5	90,9%		0,8%			8,3%			
**Murshid, 2010** (87)	Asia	1024	13-15			21,0%			15,0%			
**Ng'ang'a, 1996** (90)	Africa	919	13-15			6,0%			1,0%			
**Nguyen, 2014** (92)	Asia	200	12 & 18	67,0%		17,5%			15,5%			
**Onyeaso, 2004** (95)	Africa	636	12-17	24,5%	50,0%		12,3%	1,4%	11,8%			
**Oshagh, 2010** (96)	Asia	700	0-14		52,0%	32,6%			12,3%			
**Perillo, 2010** (98)	Europe	703	12.2*	59,5%		36,6%	13,1%	2,6%	4,3%			
**Perinetti, 2008** (99)	Europe	1198	7-11		46,8%	16,8%			6,3%			
**Rapeepattana, 2019** (101)	Asia	202	8-9	6,4%	78,7%		7,9%	3,5%	3,5%			
**Rauten, 2016** (102)	Europe	147	6 & 9	49,4%		26,6%			6,7%			
**Robke, 2007** (103)	Europe	434	2-6			21,9%			2,1%			
**Sepp, 2017** (111)	Europe	392	7.1-10.4	57,4%		21,9%			1,5%			
**Sepp, 2019** (112)	Europe	390	4-5		42,8%						33,6%	47,9%
**Sonnesen, 1998** (116)	Europe	104	7-13			72,1%			1,9%			
**Stahl, 2003** (117)	Europe	8864	2-10					4,4%				
**Steinmassl, 2017** (119)	Europe	157	8-10	64,3%		33,1%		2,5%				
**Sundareswaran, 2019** (120)	Asia	1554	13-15	74,4%		9,4%	5,9%	1,6%	8,0%			
**Sunil, 2019** (121)	Asia	100	13-17	7,0%	80,0%	8,0%			12,0%			
**Tausche, 2004** (123)	Europe	1975	6-8						3,2%			
**Thilander, 2001** (124)	America	4724	5-17			20,8%			3,7%			
**Thomaz, 2013** (125)	America	2060	12-15	13,6%		46,5%			39,1%			
**Todor, 2019** (126)	Europe	960	7-14	60,2%			21,4%	13,2%	5,2%			
**Yu, 2019** (132)	Asia	2810	7-9	43,2%		50,9%			5,9%			
**Zhou, 2017** (133)	Asia	2335	3-5							38,7%	11,3%	35,8%

**WEIGHTED MEAN**				46,3%	46,5%	25,0%	16,7%	4,7%	7,0%	41,7%	12,4%	38,5%
**WEIGHTED SD**				27,3%	17,0%	13,2%	12,7%	2,4%	7,9%	15,2%	8,1%	10,7%
**MIN**				1,7%	7,4%	0,8%	1,7%	1,4%	0,5%	18,2%	0,0%	6,0%
**MAX**				93,6%	84,0%	72,1%	40,0%	13,2%	39,1%	84,3%	33,6%	65,9%

Legend additional table S2: Prevalence of Angle classification and deciduous molar occlusion is given in percentages, along with information on the continent, the number of subjects and the age range. Angle Classification mostly recorded in permanent dentition and molar malocclusion in deciduous dentition. Only those articles included which mentioned prevalence of Angle classes and/or molar occlusion are included in this table. Furthermore the weighted mean and weighted standard deviation is given for each occlusion. Y : age range is noted, but if not available then mean +/- SD are noted, but * if SD not mentioned in article **Abbreviations**: Y: years, SD: Standard deviation, Class I: Angle Class I normal molar occlusion (well aligned dental arches without any anomalies), Class I malocclusion: Angle Class I molar occlusion but with an anomaly, Class II: Angle Class II malocclusion, Class II, 1: Angle Class II, 1 malocclusion, Class II, 2: Angle Class II,2 malocclusion, Class III: Angle Class III malocclusion, FTP: Flush distal plane second deciduous molars, DS: Distal step second deciduous molars, MS: Mesial step second deciduous.

Additional table S3: Prevalence of different transversal malocclusions and anterior crossbite.

Authors	Continent	Number Subjects	Age in Y	Crossbite (not specified)	Posterior crossbite			Anterior crossbite	Scissor bite	Forced bite/ crossbite with frontal/lateral shift
						Unilateral	Bilateral			
**Abumelha, 2018** (13)	Asia	526	6-12		12,5%	9,5%	3,0%	10,1%		
**Alajlan, 2019** (14)	Asia	520	7-12		13,3%			5,2%	4,0%	
**Al-Emran, 1990** (17)	Asia	500	13.5-14.5		7,2%				3,2%	
**Arabiun, 2014** (21)	Asia	1338	14-18		1,9%					
**Araki, 2017** (22)	Asia	420	10-16		3,8%			4,0%		
**Baral, 2014** (25)	Asia	506	3-5		0,4%			3,0%		
**Behbehani, 2005** (28)	Asia	1299	13-14		25,2%			20,8%		
**Berneburg, 2010** (29)	Europe	2015	4-6		10,7%	10,1%	0,6%			
**Bhayya, 2011** (31)	Asia	1000	4 – 6		0,3%			1,3%	0,6%	
**Bilgic, 2015** (32)	Asia	2329	12-16		9,5%	4,0%	5,5%		0,3%	
**Bourzgui, 2012** (33)	Africa	1000	8-12		7,1%	5,5%	1,6%			
**Calzada Bandomo, 2014** (34)	America	210	5 – 11		7,9%			3,4%		
**Campos-Arias, 2013** (35)	America	88	7.0*		10,1%			19,3%		
**Carvalho, 2011** (36)	America	1069	5-5y11m		13,1%			6,7%		
**Ciuffolo, 2005** (38)	Europe	810	11-14		12,2%			5,4%		
**Coetzee, 2000** (39)	Africa	214	3-8		2,8%			7,0%		
**Cosma, 2017** (40)	Europe	172	3-6		32,0%			22,0%		
**de Almeida, 2008** (43)	America	344	3.94*		11,3%	10,5%	0,8%			
**de Araújo Guimarães, 2017** (44)	America	390	8-10					0,3%		
**de Muniz, 1986** (45)	America	1554	12-13		18,0%	13,5%	4,5%		0,0%	
**Dimberg, 2015** (46)	Europe	277	3 & 7 & 11.5			11,0%	3,1%		0,7%	
**Fernandes, 2008** (50)	America	148	3-6		13,9%			1,0%		
**Ferro, 2016** (51)	Europe	380	14	10,0%		5,0%	3,0%	4,0%		
**Ferro, 2016** (52)	Europe	1960	3-5		3,7%	6,6%	0,6%	3,3%		

**Gàbris, 2006** (54)	Europe	483	16-18	11,6%		7,9%	0,0%	1,0%		
**Gois, 2012** (55)	America	212	8-11		29,2%			3,3%		
**Grabowski, 2007** (56)	Europe	3041	4.5*&8.3*		9,6%	7,7%	2,0%		0,2%	
**Gudipaneni, 2018** (58)	Asia	500	7-12		9,4%			4,8%		
**Howell, 1993** (63)	Oceania	154	13-17			13,0%	6,5%	12,0%		
**Ingervall, 1975** (64)	Europe	200	8-16	9,0%					8,0%	
**Jamilian, 2010** (65)	Asia	350	14-17		6,9%			6,0%		
**Jerez 2014** (66)	America	120	3-6		0,8%			3,3%		
**Kabue, 1995** (69)	Africa	221	3-6		1,0%			5,0%	3,0%	
**Kasparviciene, 2014** (71)	Europe	709	5-7		6,8%					21,9%
**Kolawole, 2019** (73)	Africa	992	1-12		2,1%					
**Komazaki, 2012** (74)	Asia	963	12-15		7,1%			18,6%	3,4%	
**Lux, 2009** (78)	Europe	494	8.6- 9.6		5,9%			4,3%		
**Madiraju, 2021** (79)	Asia	282	8-9		6,0%			4,3%		
**Mohamed, 2014** (84)	Asia	106	8-10	18,9%						
**Mtaya, 2009** (85)	Africa	1601	12-14		5,1%				14,3%	22,5%
**Mtaya, 2017** (86)	Africa	253	3-5	1,2%						7,9%
**Muyasa, 2012** (88)	Africa	1382	12-15					6,2%		
**Ng'ang'a, 1991** (89)	Africa	251	13-15					0,4%		
**Ng'ang'a, 1996** (90)	Africa	919	13-15		10,0%				5,0%	1,1%
**Nguyen, 2014** (92)	Asia	200	12 & 18	22,8%						
**Oshagh, 2010** (96)	Asia	700	0-14	36,0%						
**Perillo, 2010** (98)	Europe	703	12.2*			11,2%	2,9%		3,5%	
**Perinetti, 2008** (99)	Europe	1198	7-11			10,9%	3,4%	8,2%		
**Rapeepattana, 2019** (101)	Asia	202	8-9		1,0%				2,5%	19,0%
**Rauten, 2016** (102)	Europe	147	6 & 9		6,7%			9,9%		
**Robke, 2007** (103)	Europe	434	2-6		9,0%					3,0%
**Sepp, 2017** (111)	Europe	392	7.1-10.4		10,2%				1,5%	
**Sepp, 2019** (112)	Europe	390	4-5		17,4%				0,5%	
**Shalish, 2013** (113)	Asia	432	7-11		23,3%			9,5%		8,6%
**Sonnesen, 1998** (116)	Europe	104	7-13	22,1%				7,7%	2,9%	12,5%
**Stahl, 2003** (117)	Europe	8864	2-10	3,5%						
**Steinmassl, 2017** (119)	Europe	157	8-10		15,3%			14,6%	1,9%	

**Sundareswaran, 2019** (120)	Asia	1554	13-15		5,1%			7,3%	4,4%	
**Sunil, 2019** (121)	Asia	100	13-17	32,0%						
**Tausche, 2004** (123)	Europe	1975	6-8	8,2%					0,5%	13,2%
**Thilander, 2001** (124)	America	4724	5-17		4,6%				1,3%	
**Todor, 2019** (126)	Europe	960	7-14		6,7%				1,4%	
**Uematsu, 2012** (127)	Asia	2378	12-13&15-16	1,0%						
**Vithanaarchchi, 2017** (129)	Asia	721	8-15					26,7%		
**Wagner, 2015** (130)	Europe	377	3		3,4%					
**Yu, 2019** (132)	Asia	2810	7-9		2,6%			10,5%	0,9%	
**Zhou, 2017** (133)	Asia	2335	3-5		0,3%			8,0%		
Weighted Mean				6,2%	7,6%	8,3%	2,5%	8,3%	2,2%	13,7%
Weighted Standard Deviation				7,8%	6,0%	2,9%	1,8%	6,0%	3,4%	7,7%
minimum				1,0%	0,3%	4,0%	0,0%	0,3%	0,0%	1,1%
maximum				36,0%	32,0%	13,5%	6,5%	26,7%	14,3%	22,5%

Legend additional table S3: Prevalence of different transversal malocclusions: crossbite (not specified, posterior crossbite, unilateral- and bilateral crossbite, anterior crossbite, scissor bite and crossbite with functional shift is given in percentages, along with information on the continent, the number of subjects and the age range. Y : age range is noted, but if not available then mean +/- SD are noted, but * if SD not mentioned in article. **Abbreviations**: Y: years, SD: Standard deviation, CB: cross bite

Additional table S4: Prevalence of tooth anomalies.

Authors	Continent	Number Subjects	Age in Y	Agenesis / Hypodontia	Mesiodens	Super-numerary teeth / Hyper-dontia	Hypo-Hyper-dontia	Impacted / retained teeth (Impeded eruption)	Ectopic eruption	Trans-position
**Aasheim, 1993** (11)	Europe	1953	9	6,5%						
**Abu Alhaija, 2005** (12)	Asia	1003	13-15	6,0%				1,8%	5,9%	0,3%
**Al-Amiri, 2013** (15)	America	496	16y3m*	9,5%		1,4%		12,9%		
**Alberti, 2006** (16)	Europe	1577	6-10		0,3%	0,4%				
**al-Emran, 1990** (17)	Asia	500	13.5-14.5	4,0%				10,4%	4,4%	0,8%
**Alkilzy, 2007** (18)	Asia	234	2-16	4,3%		3,8%		6,4%		
**Alsoleihat, 2014** (19)	Asia	85	14-18	11,8%						
**Altug-Atac, 2005** (20)	Asia	3043	8.5 - 14.75	2,6%						
**Araki, 2017** (22)	Asia	420	10-16	9,0%						
**Baccetti, 1998** (23)	Europe	5450	7-14			3,9%				
**Badrov, 2017** (24)	Europe	4430	6-15	7,2%						
**Baron, 2018** (26)	Europe	551	15.23*	5,8%		1,1%		2,7%		0,4%
**Bhardwaj, 2011** (30)	Asia	622	16-17	8,0%						
**Bourzgui, 2012** (33)	Africa	1000	8-12	1,5%		0,2%			9,7%	0,1%
**Calzada Bandomo, 2014** (34)	America	210	5 – 11	4,8%				1,5%		
**Chauhan, 2013** (37)	Asia	1188	9-12	0,7%						
**Daou, 2019** (42)	Asia	334	7.31 +/- 2.17	8,7%		0,3%				
**Endo, 2006** (47)	Asia	3358	5-15	18,6%						
**Esenlik, 2009** (49)	Asia	2599	6-16			2,7%				
**Frazao, 2006** (53)	America	13801	12 & 18	4,0%						
**Gracco, 2017** (57)	Europe	4006	9-16	8,9%						
**Guttierez Marin, 2019** (59)	America	157	6-12	3,8%		4,5%				
**Harris, 2008** (60)	America	1700	12-18	16,5%						
**Harris, 2008** (61)	America	1700	12-18			2,3%				

**Howell, 1993** (63)	Oceania	154	13-17	7,0%		1,0%		5,0%		
**Ingervall, 1975** (64)	Europe	200	8-16	17,5%						
**Jamilian, 2010** (65)	Asia	350	14-17					7,1%		
**Johannsdottir, 1997** (67)	Europe	396	6	5,0%						
**Kabue, 1995** (69)	Africa	221	3-6	4,0%		1,0%				
**Kielan-Grabowska, 2019** (72)	Europe	674	6-15	11,6%						
**Komazaki, 2012** (74)	Asia	963	12-15	0,0%						
**Lagana, 2017** (76)	Europe	4706	8-12	7,1%		0,9%		3,9%	7,5%	1,4%
**Lara, 2013** (77)	America	1995	4-13		1,5%	1,5%				
**Medina, 2012** (83)	America	607	5-11	4,0%						
**Ng'ang'a, 1996** (90)	Africa	919	13-15			0,2%		3,0%		0,3%
**Ng'ang'a, 2001** (91)	Africa	615	8-15Y	6,3%						
**O' Dowling, 1989** (93)	Europe	3056	7-17				0,5%			
**O' Dowling, 1990** (94)	Europe	3056	7-17	11,3%						
**Pagan- Collazo, 2014** (97)	America	1911	10-14	6,0%						
**Pineda, 2011** (100)	America	307	6-11	4,2%						
**Rapeepattana, 2019** (101)	Asia	202	8-9	1,5%		1,0%		0,5%		
**Rolling, 1980** (104)	Europe	3325	9-10	7,8%						
**Rozsa, 2009** (105)	Europe	4417	6-18	0.29 %						
**Sejdini, 2018** (110)	Europe	520	7 - 14	3,5%		0,8%				
**Shalish, 2013** (113)	Asia	432	7-11						0,9%	
**Sola, 2018** (115)	Europe	2500	7-11	3,5%						
**Sonnesen, 1998** (116)	Europe	104	7-13	13.5 %						
**Stahl, 2003** (118)	Europe	4208	6.7- 13.4	9,2%	1,6%	2,8%				
**Sundareswaran, 2019** (120)	Asia	1554	13-15	6,6%					11,1%	
**Swarnalatha, 2020** (122)	Asia	1000	12-18	3.77 %						
**Thilander, 2001** (124)	America	4724	5-17	3,2%		1,8%		3,1%	1,5%	
**Varela, 2009** (128)	Europe	2108	7-16	6,5%		2,0%	0,3%			
**Yassin, 2016** (131)	Asia	1252	5-12	9,7%		3,5%			2,3%	
Weighted Mean				6,5%	1,3%	2,1%	0,4%	4,0%	5,3%	0,9%
Weighted Standard Deviation				4,2%	0,5%	1,2%	0,1%	2,4%	3,5%	0,6%

Minimum				0,0%	0,3%	0,2%	0,3%	0,5%	0,9%	0,1%
Maximum				18,6%	1,6%	4,5%	0,5%	12,9%	11,1%	1,4%

Legend additional table S4: Prevalence of tooth anoamlies: hypodontia, hyperdontia, hypo-hyperdontia, impacted/retained teeth, ectopic eruption, transposition are noted as in percentages is given in percentages, along with information on the continent, the number of subjects and the age range. Y : age range is noted, but if not available then mean +/- SD are noted, but * if SD not mentioned in article. . Y : age range is noted, but if not available then mean +/- SD are noted, but * if SD not mentioned in article **Abbreviations**: Y: years, SD: Standard deviation

Additional table S5: Prevalence of space anomalies

Authors	Continent	Number Subjects	Age in Y	Crowding maxillary arch	Crowding mandibular arch	Crowding	Spacing maxillary arch	Spacing mandibular arch	Spacing	Midline diastema
**Abu Alhaija, 2005** (12)	Asia	1003	13-15			50,4%				
**Abumelha, 2018** (13)	Asia	526	6-12			36,5%				
**al-Emran, 1990** (17)	Asia	500	13.5-14.5	19,4%	23,4%		17,0%	8,0%		3,6%
**Alkilzy, 2007** (18)	Asia	234	2-16							
**Araki, 2017** (22)	Asia	420	10-16			11,9%				
**Baskaradoss, 2013** (27)	Asia	300	11-15			41,2%			12,4%	7,4%
**Bhardwaj, 2011** (30)	Asia	622	16-17			62,4%			29,6%	
**Bhayya, 2011** (31)	Asia	1000	4 – 6	1,7%	4,6%					
**Bilgic, 2015** (32)	Asia	2329	12-16			65,2%			1,8%	
**Calzada Bandomo, 2014** (34)	America	210	5 – 11			9,3%				
**Campos-Arias, 2013** (35)	America	88	7.0*			48,9%				
**Chauhan, 2013** (37)	Asia	1188	9-12			17,8%			1,5%	
**Ciuffolo, 2005** (38)	Europe	810	11-14			20,2%			5,6%	
**Dacosta, 1999** (41)	Africa	1028	11-18	21,7%	36,3%		45,9%	30,0%		
**de Almeida, 2008** (43)	America	344	3.94*	7,0%	11,3%					
**de Araújo Guimarães, 2018** (44)	America	390	8-10	19,7%	10,5%				44,9%	3,6%
**de Muniz, 1986** (45)	America	1554	12-13	6,2%	6,7%		1,8%	1,3%		
**Dimberg, 2015** (46)	Europe	277	3 & 7 & 11.5			31,0%			9,4%	6,5%
**Esa, 2001** (48)	Asia	1519	12-13	40,6%	22,2%				17,4%	
**Ferro, 2016** (51)	Europe	380	14	17,0%	19,0%	30,0%				1,0%
**Ferro, 2016** (52)	Europe	1960	3-5							
**Frazao, 2006** (53)	America	13801	12 & 18	18,9%	12,9%	38,0%			21,4%	
**Gàbris, 2006** (54)	Europe	483	16-18						7,8%	
**Gois, 2012** (55)	America	212	8-11			34,9%				
**Gudipaneni, 2018** (58)	Asia	500	7-12			47,2%			27,2%	
**Hassanali, 1993** (62)	Africa	412	3-16						44,4%	
**Howell, 1993** (63)	Oceania	154	13-17			72,0%			17,5%	

**Ingervall, 1975** (64)	Europe	200	8-16	14,0%	12,0%		21,0%	10,5%		
**Jamilian, 2010** (65)	Asia	350	14-17			93,4%				
**Johannsdottir, 1997** (67)	Europe	396	6	77,9%	83,3%				42,5%	
**Johnson, 2000** (68)	Oceania	294	9.9 - 11. 3	6,0%		80,3%			59,5%	
**Kabue, 1995** (69)	Africa	221	3-6	4,0%	10,0%					
**Kasparviciene, 2014** (71)	Europe	709	5-7						52,2%	
**Kolawole, 2019** (73)	Africa	992	1-12			21,7%			29,9%	
**Komazaki, 2012** (74)	Asia	963	12-15	67,4%	67,9%					2,9%
**Madiruja, 2021** (79)	Asia	282	8-9			39,7%			7,1%	
**Mail, 2015** (80)	America	50	12		48,0%				36,0%	28,0%
**Martins, 2009** (81)	America	264	10-12	6,1%	16,3%	40,1%				14,8%
**Martins, 2019** (82)	America	1612	11-14			51,9%			32,1%	23,7%
**Mohamed, 2014** (84)	Asia	106	8-10	24,5%	57,5%		62,2%	23,6%		
**Mtaya, 2009** (85)	Africa	1601	12-14			14,1%			21,9%	
**Mtaya, 2017** (86)	Africa	253	3-5			0,8%			19,8%	
**Murshid, 2010** (87)	Asia	1024	13-15	39,0%	58,0%					
**Muyasa, 2012** (88)	Africa	1382	12-15	38,6%	31,1%	47,2%			46,6%	
**Ng'ang'a, 1991** (89)	Africa	251	13-15	25,4%						
**Ng'ang'a, 1996** (90)	Africa	919	13-15	21,0%	18,0%		17,0%	13,0%		
**Nguyen, 2014** (92)	Asia	200	12 & 18			54,0%				
**Onyeaso, 2004** (95)	Africa	636	12-17	5,2%	7,7%	7,2%				36,8%
**Perillo, 2010** (98)	Europe	703	12.2*			45,9%			22,9%	
**Perinetti, 2008** (99)	Europe	1198	7-11	4,0%	23,2%	17,2%				
**Robke, 2007** (103)	Europe	434	2-6			36,9%				
**Rwakatema, 2007** (106)	Africa	289	12-15			41,2%			28,4%	
**Sanadhya, 2014** (107)	Asia	947	12-15			40,2%			27,1%	15,3%
**Sánchez-Pérez, 2013** (108)	America	249	15			50,0%			7,2%	
**Seemann, 2011** (109)	Europe	2975	4 & 7.8*			30,3%				
**Sepp, 2017** (111)	Europe	392	7.1-10.4	18,9%	37,9%	49,7%	57,7%	15,3%		73,0%
**Sepp, 2019** (112)	Europe	390	4-5	0,0%	0,3%					34,9%
**Singh, 2011** (114)	Asia	927	12			45,4%			10,0%	9,8%
**Sonnesen, 1998** (116)	Europe	104	7-13			56,7%			13,5%	
**Stahl, 2003** (117)	Europe	8864	2-10			10,1%			1,2%	
**Steinmassl, 2017** (119)	Europe	157	8-10	22,3%	31,8%		38,9%	17,2%		
**Sundareswaran, 2019** (120)	Asia	1554	13-15			66,6%			15,0%	
**Sunil, 2019** (121)	Asia	100	13-17			70,0%				6,0%

**Tausche, 2004** (123)	Europe	1975	6-8	12,0%	14,3%					
**Thilander, 2001** (124)	America	4724	5-17			52,1%			25,9%	7,0%
**Todor, 2019** (126)	Europe	960	7-14			47,5%			3,5%	
**Uematsu, 2012** (127)	Asia	2378	12-13 & 15-16			19,9%				
**Yu, 2019** (132)	Asia	2810	7-9			28,4%			9,5%	
**Zhou, 2017** (133)	Asia	2335	3-5			6,5%			44,8%	
Weighted Mean				20,8%	19,7%	33,8%	23,4%	12,8%	18,7%	13,8%
Weighted Standard Deviation				14,5%	15,8%	18,1%	20,1%	10,6%	13,7%	14,2%
Minimum				1,7%	0,3%	0,8%	1,8%	1,3%	1,2%	1,0%
Maximum				77,9%	83,3%	93,4%	62,2%	30,0%	59,5%	73,0%

Legend additional table S5: Prevalence of space anomalies: crowding, spacing and midline diastema are noted as in percentages, along with information on the continent, the number of subjects and the age range. Y: age range is noted, but if not available then mean +/- SD are noted, but * if SD not mentioned in article **Abbreviations**: Y: years;

Additional table S6: Prevalence of Angle classification and deciduous molar malocclusion according to geographical location.

Authors	Continent	Number Subjects	Age in Y	Class I	Class I mal-occlusion	Class II	Class II, 1	Class II,2	Class III	FTP	DS	MS
**Bourzgui, 2012** (33)	Africa	1000	8-12	61,4%		24,0%			10,0%			
**Coetzee, 2000** (39)	Africa	214	3-8							18,2%	1,9%	65,9%
**Dacosta, 1999** (41)	Africa	1028	11-18	12,3%	84,0%		1,7%		2,1%			
**Kabue, 1995** (69)	Africa	221	3-6							53,0%	0,0%	44,0%
**Mtaya, 2009** (85)	Africa	1601	12-14	93,6%		4,4%			2,0%			
**Mtaya, 2017** (86)	Africa	253	3-5	90,9%		0,8%			8,3%			
**Ng'ang'a, 1996** (90)	Africa	919	13-15			6,0%			1,0%			
**Onyeaso, 2004** (95)	Africa	636	12-17	24,5%	50,0%		12,3%	1,4%	11,8%			
Weighted Mean				58,1%	71,0%	9,7%	5,8%	1,4%	4,8%	35,9%	0,9%	54,8%
Weighted Standard Deviation				33,9%	16,5%	8,6%	5,2%	0,0%	4,2%	17,4%	1,0%	11,0%
Minimum				12,3%	50,0%	0,8%	1,7%	1,4%	1,0%	18,2%	0,0%	44,0%
Maximum				93,6%	84,0%	24,0%	12,3%	1,4%	11,8%	53,0%	1,9%	65,9%
**Campos-Arias, 2013** (35)	America	88	7.0*			10,2%			11,3%			
**de Almeida, 2008** (43)	America	344	3.94*							84,3%	9,7%	6,0%
**Gois, 2012** (55)	America	212	8-11	8,5%	54,2%	28,3%			9,0%			
**Jerez 2014** (66)	America	120	3-6	1,7%						44,1%	2,9%	44,1%
**Martins, 2009** (82)	America	264	10-12	25,8%	47,7%		17,0%	5,3%	4,2%			
**Thilander, 2001** (124)	America	4724	5-17			20,8%			3,7%			
**Thomaz, 2013** (125)	America	2060	12-15	13,6%		46,5%			39,1%			
Weighted Mean				13,9%	50,6%	28,4%	17,0%	5,3%	13,9%	73,9%	7,9%	15,9%
Weighted Standard Deviation				4,8%	3,2%	11,7%	0,0%	0,0%	15,8%	17,6%	3,0%	16,7%
Minimum				1,7%	47,7%	10,2%	17,0%	5,3%	3,7%	44,1%	2,9%	6,0%
Maximum				25,8%	54,2%	46,5%	17,0%	5,3%	39,1%	84,3%	9,7%	44,1%

**Abu Alhaija, 2005** (12)	Asia	1003	13-15		55,3%	17,5%			1,5%			
**Abumelha, 2018** (13)	Asia	526	6-12		65,0%		12,9%	1,7%	8,4%			
**Alajlan, 2019** (14)	Asia	520	7-12		7,4%	32,3%			8,3%			
**Al-Emran, 1990** (17)	Asia	500	13.5-14.5			16,4%			3,0%			
**Arabiun, 2014** (21)	Asia	1338	14-18	74,7%	12,7%	9,9%	4,8%	5,2%	1,0%			
**Baral, 2014** (25)	Asia	506	3-5							33,5%	8,5%	40,3%
**Behbehani, 2005** (28)	Asia	1299	13-14		57,8%	31,2%			11,0%			
**Bhayya, 2011** (31)	Asia	1000	4 – 6							52,5%	8,4%	35,9%
**Bilgic, 2015** (32)	Asia	2329	12-16	10,1%	34,9%		40,0%	4,7%	10,3%			
**Chauhan, 2013** (37)	Asia	1188	9-12	90,9%								
**Gudipaneni, 2018** (58)	Asia	500	7-12	52,8%		31,8%			15,4%			
**Kalbassi, 2019** (70)	Asia	1208	7-15		46,0%	36,3%			4,4%			
**Komazaki, 2012** (74)	Asia	963	12-15	51,3%		12,3%	18,5%	7,5%	10,5%			
**Madiraju, 2021** (79)	Asia	282	8-9	75,2%		23,4%			1,4%			
**Mohamed, 2014** (84)	Asia	106	8-10	39,6%		49,1%			11,3%			
**Murshid, 2010** (87)	Asia	1024	13-15			21,0%			15,0%			
**Nguyen, 2014** (92)	Asia	200	12 & 18	67,0%		17,5%			15,5%			
**Oshagh, 2010** (96)	Asia	700	0-14		52,0%	32,6%			12,3%			
**Rapeepattana, 2019** (101)	Asia	202	8-9	6,4%	78,7%		7,9%	3,5%	3,5%			
**Sundareswaran, 2019** (120)	Asia	1554	13-15	74,4%		9,4%	5,9%	1,6%	8,0%			
**Sunil, 2019** (121)	Asia	100	13-17	7,0%	80,0%	8,0%			12,0%			
**Yu, 2019** (132)	Asia	2810	7-9	43,2%		50,9%			5,9%			
**Zhou, 2017** (133)	Asia	2335	3-5							38,7%	11,3%	35,8%
Weighted Mean				50,6%	41,5%	27,4%	19,5%	4,2%	7,8%	41,6%	10,2%	36,4%
Weighted Standard Deviation				26,9%	18,5%	14,9%	15,2%	1,9%	4,2%	6,7%	1,4%	1,5%
Minimum				6,4%	7,4%	8,0%	4,8%	1,6%	1,0%	33,5%	8,4%	35,8%
Maximum				90,9%	80,0%	50,9%	40,0%	7,5%	15,5%	52,5%	11,3%	40,3%

**Cosma, 2017** (40)	Europe	172	3-6							58,0%	5,0%	36,0%
**Dimberg, 2015** (46)	Europe	277	3 & 7 & 11.5		74,7%	20,9%	5,7%	1,8%	4,4%			
**Ferro, 2016** (51)	Europe	380	14	86,3%		19,5%			3,9%			

**Gàbris, 2006** (54)	Europe	483	16-18	52,8%								
**Grabowski, 2007** (56)	Europe	3041	4.5* & 8.3*		44,7%	28,6%			2,6%			
**Ingervall, 1975** (64)	Europe	200	8-16			14,5%			0,5%			
**Kasparviciene, 2014** (71)	Europe	709	5-7	10,1%		10,6%			0,8%	20,9%	24,9%	50,2%
**Lagana, 2013** (75)	Europe	2617	7-15	40,4%		29,2%			3,2%			
**Lux, 2009** (78)	Europe	494	8.6- 9.6			10,2%			3,3%			
**Perillo, 2010** (98)	Europe	703	12.2*	59,5%		36,6%	13,1%	2,6%	4,3%			
**Perinetti, 2008** (99)	Europe	1198	7-11		46,8%	16,8%			6,3%			
**Rauten, 2016** (102)	Europe	147	6 & 9	49,4%		26,6%			6,7%			
**Robke, 2007** (103)	Europe	434	2-6			21,9%			2,1%			
**Sepp, 2017** (111)	Europe	392	7.1-10.4	57,4%		21,9%			1,5%			
**Sepp, 2019** (112)	Europe	390	4-5		42,8%						33,6%	47,9%
**Sonnesen, 1998** (116)	Europe	104	7-13			72,1%			1,9%			
**Stahl, 2003** (117)	Europe	8864	2-10					4,4%				
**Steinmassl, 2017** (119)	Europe	157	8-10	64,3%		33,1%		2,5%				
**Tausche, 2004** (123)	Europe	1975	6-8						3,2%			
**Todor, 2019** (126)	Europe	960	7-14	60,2%			21,4%	13,2%	5,2%			
Weighted Mean				47,4%	46,8%	25,1%	16,1%	4,9%	3,4%	28,1%	24,9%	47,6%
Weighted Standard Deviation				17,7%	6,9%	8,6%	5,7%	2,6%	2,6%	14,7%	8.8.%	4,7%
Minimum				10,1%	42,8%	10,2%	5,7%	1,8%	0,5%	20,9%	5,0%	36,0%
Maximum				86,3%	74,7%	72,1%	21,4%	13,2%	6,7%	58,0%	33,6%	50,2%

**Howell, 1993** (63)	Oceania	154	13-17	65,0%			15,0%	12,0%	7,0%			
Weighted Mean				65,0%			15,0%	12,0%	7,0%			
Weighted Standard Deviation				0,0%			0,0%	0,0%	0,0%			
Minimum				65,0%			15,0%	12,0%	7,0%			
Maximum				65,0%			15,0%	12,0%	7,0%			

Legend additional table S6: Prevalence of Angle classification and deciduous molar occlusion is given in percentages according to geographical location (continent), along with information on the continent, the number of subjects and the age range. Angle Classification mostly recorded in permanent dentition and molar malocclusion in deciduous dentition. Only those articles included which mentioned prevalence of Angle classes and/or molar occlusion are included in this table. Furthermore the weighted mean and weighted standard deviation is given for each occlusion. Y : age range is noted, but if not available then mean +/- SD are noted, but * if SD not mentioned in article
Abbreviations: Y: years, SD: Standard deviation, Class I: Angle Class I normal molar occlusion (well aligned dental arches without any anomalies), Class I malocclusion: Angle Class I molar occlusion but with an anomaly, Class II: Angle Class II malocclusion, Class II, 1: Angle Class II, 1 malocclusion, Class II, 2: Angle Class II,2 malocclusion, Class III: Angle Class III malocclusion, FTP: Flush distal plane second deciduous molars, DS: Distal step second deciduous molars, MS: Mesial step second deciduous.

Additional table S7: Prevalence of different transversal malocclusions and anterior crossbite according to geographical location.

Authors	Continent	Number Subjects	Age in Y	Crossbite (not specified)	Posterior crossbite	Posterior crossbite unilateral	Posterior crossbite bilateral	Anterior crossbite	Scissor bite	Forced bite/ crossbite with frontal/ lateral shift
**Bourzgui, 2012** (33)	Africa	1000	8-12		7,1%	5,5%	1,6%			
**Coetzee, 2000** (39)	Africa	214	3-8		2,8%			7,0%		
**Kabue, 1995** (69)	Africa	221	3-6		1,0%			5,0%	3,0%	
**Kolawole, 2019** (73)	Africa	992	1-12		2,1%					
**Mtaya, 2009** (85)	Africa	1601	12-14		5,1%				14,3%	22,5%
**Mtaya, 2017** (86)	Africa	3	3-5	1,2%						7,9%
**Muyasa, 2012** (88)	Africa	1382	12-15					6,2%		
**Ng'ang'a, 1991** (89)	Africa	251	13-15					0,4%		
**Ng'ang'a, 1996** (90)	Africa	919	13-15		10,0%				5,0%	1,1%
Weighted Mean				1,2%	5,5%	5,5%	1,6%	5,5%	10,3%	14,7%
Weighted Standard Deviation				0,0%	2,8%	0,0%	0,0%	1,9%	4,8%	10,3%
Minimum				1,2%	1,0%	5,5%	1,6%	0,4%	3,0%	1,1%
Maximum				1,2%	10,0%	5,5%	1,6%	7,0%	14,3%	22,5%

**Calzada Bandomo, 2014** (34)	America	210	5 – 11		7,9%			3,4%		
**Campos-Arias, 2013** (35)	America	88	7.0*		10,1%			19,3%		
**Carvalho, 2011** (36)	America	1069	5-5y11m		13,1%			6,7%		
**de Almeida, 2008** (43)	America	344	3.94*		11,3%	10,5%	0,8%			
**de Araújo Guimarães, 2017** (44)	America	390	8-10					0,3%		
**de Muniz, 1986** (45)	America	1554	12-13		18,0%	13,5%	4,5%		0,0%	
**Fernandes, 2008** (50)	America	148	3-6		13,9%			1,0%		
**Gois, 2012** (55)	America	212	8-11		29,2%			3,3%		

**Jerez 2014** (66)	America	120	3-6		0,8%			3,3%		
**Thilander, 2001** (124)	America	4724	5-17		4,6%				1,3%	
Weighted Mean					9,3%	13,0%	3,8%	4,9%	1,0%	
Weighted Standard Deviation					6,3%	1,2%	1,4%	3,9%	0,6%	
Minimum					0,8%	10,5%	0,8%	0,3%	0,0%	
Maximum					29,2%	13,5%	4,5%	19,3%	0,6%	

**Abumelha, 2018** (13)	Asia	526	6-12		12,5%	9,5%	3,0%	10,1%		
**Alajlan, 2019** (14)	Asia	520	7-12		13,3%			5,2%	4,0%	
**Al-Emran, 1990** (17)	Asia	500	13.5-14.5		7,2%				3,2%	
**Arabiun, 2014** (21)	Asia	1338	14-18		1,9%					
**Araki, 2017** (22)	Asia	420	10-16		3,8%			4,0%		
**Baral, 2014** (25)	Asia	506	3-5		0,4%			3,0%		
**Behbehani, 2005** (28)	Asia	1299	13-14		25,2%			20,8%		
**Bhayya, 2011** (31)	Asia	1000	4 – 6		0,3%			1,3%	0,6%	
**Bilgic, 2015** (32)	Asia	2329	12-16		9,5%	4,0%	5,5%		0,3%	
**Gudipaneni, 2018** (58)	Asia	500	7-12		9,4%			4,8%		
**Jamilian, 2010** (65)	Asia	350	14-17		6,9%			6,0%		
**Komazaki, 2012** (74)	Asia	963	12-15		7,1%			18,6%	3,4%	
**Madiraju, 2021** (79)	Asia	282	8-9		6,0%			4,3%		
**Mohamed, 2014** (84)	Asia	106	8-10	18,9%						
**Nguyen, 2014** (92)	Asia	200	12 & 18	22,8%						
**Oshagh, 2010** (96)	Asia	700	0-14	36,0%						
**Rapeepattana, 2019** (101)	Asia	202	8-9		1,0%				2,5%	19,0%
**Shalish, 2013** (113)	Asia	432	7-11		23,3%			9,5%		8,6%
**Sundareswaran, 2019** (120)	Asia	1554	13-15		5,1%			7,3%	4,4%	
**Sunil, 2019** (121)	Asia	100	13-17	32,0%						
**Uematsu, 2012** (127)	Asia	2378	12-13 & 15-16	1,0%						
**Vithanaarchchi, 2017** (129)	Asia	721	8-15					26,7%		
**Yu, 2019** (132)	Asia	2810	7-9		2,6%			10,5%	0,9%	
**Zhou, 2017** (133)	Asia	2335	3-5		0,3%			8,0%		
Weighted Mean				8,9%	6,6%	5,0%	5,0%	10,3%	1,8%	11,9%

Weighted Standard Deviation				14,0%	7,0%	2,1%	1,0%	6,5%	1,6%	4,8%
Minimum				1,0%	0,3%	4,0%	3,0%	1,3%	0,3%	8,6%
Maximum				36,0%	25,2%	9,5%	5,5%	26,7%	4,4%	19,0%

**Berneburg, 2010** (29)	Europe	2015	4-6		10,7%	10,1%	0,6%			
**Ciuffolo, 2005** (38)	Europe	810	11-14		12,2%			5,4%		
**Cosma, 2017** (40)	Europe	172	3-6		32,0%			22,0%		
**Dimberg, 2015** (46)	Europe	277	3 & 7 & 11.5			11,0%	3,1%		0,7%	
**Ferro, 2016** (51)	Europe	380	14	10,0%		5,0%	3,0%	4,0%		
**Ferro, 2016** (52)	Europe	1960	3-5		3,7%	6,6%	0,6%	3,3%		
**Gàbris, 2006** (54)	Europe	483	16-18	11,6%		7,9%	0,0%	1,0%		
**Grabowski, 2007** (56)	Europe	3041	4.5* & 8.3*		9,6%	7,7%	2,0%		0,2%	
**Ingervall, 1975** (64)	Europe	200	8-16	9,0%					8,0%	
**Kasparviciene, 2014** (71)	Europe	709	5-7		6,8%					21,9%
**Lux, 2009** (78)	Europe	494	8.6- 9.6		5,9%			4,3%		
**Perillo, 2010** (98)	Europe	703	12.2*			11,2%	2,9%		3,5%	
**Perinetti, 2008** (99)	Europe	1198	7-11			10,9%	3,4%	8,2%		
**Rauten, 2016** (102)	Europe	147	6 & 9		6,7%			9,9%		
**Robke, 2007** (103)	Europe	434	2-6		9,0%					3,0%
**Sepp, 2017** (111)	Europe	392	7.1-10.4		10,2%				1,5%	
**Sepp, 2019** (112)	Europe	390	4-5		17,4%				0,5%	
**Sonnesen, 1998** (116)	Europe	104	7-13	22,1%				7,7%	2,9%	12,5%
**Stahl, 2003** (117)	Europe	8864	2-10	3,5%						
**Steinmassl, 2017** (119)	Europe	157	8-10		15,3%			14,6%	1,9%	
**Tausche, 2004** (123)	Europe	1975	6-8	8,2%					0,5%	13,2%
**Todor, 2019** (126)	Europe	960	7-14		6,7%				1,4%	
**Wagner, 2015** (130)	Europe	377	3		3,4%					
Weighted Mean				5,1%	8,9%	8,6%	1,6%	5,6%	1,0%	13,7%
Weighted Standard Deviation				2,9%	4,3%	1,8%	1,1%	4,0%	1,5%	5,5%
Minimum				3,5%	3,4%	5,0%	0,0%	1,0%	0,2%	3,0%
Maximum				22,1%	32,0%	11,2%	3,4%	22,0%	8,0%	21,9%

**Howell, 1993** (63)	Oceania	154	13-17			13,0%	6,5%	12,0%		

Weighted Mean						13,0%	6,5%	12,0%		
Weighted Standard Deviation						0,0%	0,0%	0,0%		
Minimum						13,0%	6,5%	12,0%		
Maximum						13,0%	6,5%	12,0%		

Legend additional table S7: Prevalence of different transversal malocclusions: crossbite (not specified, posterior crossbite, unilateral- and bilateral crossbite, anterior crossbite, scissor bite and crossbite with functional shift according to geographical location, is given in percentages, along with information on the continent, the number of subjects and the age range. Y : age range is noted, but if not available then mean +/- SD are noted, but * if SD not mentioned in article.
Abbreviations: Y: years.

Additional table S8: Prevalence of tooth anomalies according to geographical location.

Authors	Continent	Number Subjects	Age in Y	Agenesis / Hypodontia	Mesiodens	Super-numerary teeth / Hyper-dontia	Hypo-Hyper-dontia	Impacted / retained teeth (Impeded eruption)	Ectopic eruption	Trans-position
**Bourzgui, 2012** (33)	Africa	1000	8-12	1,5%		0,2%			9,7%	0,1%
**Kabue, 1995** (69)	Africa	221	3-6	4,0%		1,0%				
**Ng'ang'a, 1996** (90)	Africa	919	13-15			0,2%		3,0%		0,3%
**Ng'ang'a, 2001** (91)	Africa	615	8-15Y	6,3%						
Weighted Mean				3,4%		0,3%		3,0%	9,7%	0,2%
Weighted Standard Deviation				2,2%		0,2%		0,0%	0,0%	0,1%
Minimum				1,5%		0,2%		3,0%	9,7%	0,1%
Maximum				6,3%		1,0%		3,0%	9,7%	0,3%
**Al-Amiri, 2013** (15)	America	496	16y3m*	9,5%		1,4%		12,9%		
**Calzada Bandomo, 2014** (34)	America	210	5 – 11	4,8%				1,5%		
**Frazao, 2006** (53)	America	13801	12 & 18	4,0%						
**Guttierez Marin, 2019** (59)	America	157	6-12	3,8%		4,5%				
**Harris, 2008** (60)	America	1700	12-18	16,5%						
**Harris, 2008** (61)	America	1700	12-18			2,3%				
**Lara, 2013** (77)	America	1995	4-13		1,5%	1,5%				
**Medina, 2012** (83)	America	607	5-11	4,0%						
**Pagan- Collazo, 2014** (97)	America	1911	10-14	6,0%						
**Pineda, 2011** (100)	America	307	6-11	4,2%						
**Thilander, 2001** (124)	America	4724	5-17	3,2%		1,8%		3,1%	1,5%	
Weighted Mean				5,0%	1,5%	1,9%		3,9%	1,5%	
Weighted Standard Deviation				3,3%	0,0%	0,4%		2,9%	0,0%	

Minimum				3,2%	1,5%	1,4%		1,5%	1,5%	
Maximum				16,5%	1,5%	4,5%		12,9%	1,5%	
**Abu Alhaija, 2005** (12)	Asia	1003	13-15	6,0%				1,8%	5,9%	0,3%
**Al-Emran, 1990** (17)	Asia	500	13.5-14.5	4,0%				10,4%	4,4%	0,8%
**Alkilzy, 2007** (18)	Asia	234	2-16	4,3%		3,8%		6,4%		
**Alsoleihat, 2014** (19)	Asia	85	14-18	11,8%						
**Altug-Atac, 2005** (20)	Asia	3043	8.5 - 14.75	2,6%						
**Araki, 2017** (22)	Asia	420	10-16	9,0%						
**Bhardwaj, 2011** (30)	Asia	622	16-17	8,0%						
**Chauhan, 2013** (37)	Asia	1188	9-12	0,7%						
**Daou, 2019** (42)	Asia	334	7.31 +/- 2.17	8,7%		0,3%				
**Endo, 2006** (47)	Asia	3358	5-15	18,6%						
**Esenlik, 2009** (49)	Asia	2599	6-16			2,7%				
**Jamilian, 2010** (65)	Asia	350	14-17					7,1%		
**Komazaki, 2012** (74)	Asia	963	12-15	0,0%						
**Rapeepattana, 2019** (101)	Asia	202	8-9	1,5%		1,0%		0,5%		
**Shalish, 2013** (113)	Asia	432	7-11						0,9%	
**Sundareswaran, 2019** (120)	Asia	1554	13-15	6,6%					11,1%	
**Swarnalatha, 2020** (122)	Asia	1000	12-18	3,8%						
**Yassin, 2016** (131)	Asia	1252	5-12	9,7%		3,5%			2,3%	
Weighted Mean				8,1%		2,7%		4,8%	6,0%	0,5%
Weighted Standard Deviation				6,3%		1,6%		4,1%	4,0%	0,4%
Minimum				0,0%		0,3%		0,5%	0,9%	0,3%
Maximum				18,6%		3,8%		10,4%	11,1%	0,8%
**Aasheim, 1993** (11)	Europe	1953		6,5%						
**Alberti, 2006** (16)	Europe	1577	6-10		0,3%	0,4%				
**Baccetti, 1998** (23)	Europe	5450	7-14			3,9%				
**Badrov, 2017** (24)	Europe	4430	6-15	7,2%						
**Baron, 2018** (26)	Europe	551	15.23*	5,8%		1,1%		2,7%		0,4%

**Gracco, 2017** (57)	Europe	4006	9-16	8,9%						
**Ingervall, 1975** (64)	Europe	200	8-16	17,5%						
**Johannsdottir, 1997** (67)	Europe	396	6	5,0%						
**Kielan-Grabowska, 2019** (72)	Europe	674	6-15	11,6%						
**Lagana, 2017** (76)	Europe	4706	8-12	7,1%		0,9%		3,9%	7,5%	1,4%
**O' Dowling, 1989** (93)	Europe	3056	7-17				0,5%			
**O' Dowling, 1990** (94)	Europe	3056	7-17	11,3%						
**Rolling, 1980** (104)	Europe	3325	9-10	7,8%						
**Rozsa, 2009** (105)	Europe	4417	6-18	0.29 %						
**Sejdini, 2018** (110)	Europe	520	7 - 14	3,5%		0,8%				
**Sola, 2018** (115)	Europe	2500	7-11	3,5%						
**Sonnesen, 1998** (116)	Europe	104	7-13	13.5 %						
**Stahl, 2003** (118)	Europe	4208	6.7- 13.4	9,2%	1,6%	2,8%				
**Varela, 2009** (128)	Europe	2108	7-16	6,5%		2,0%	0,3%			
Weighted Mean				6,9%	1,3%	2,3%	0,4%	3,8%	7,5%	1,3%
Weighted Standard Deviation				3,2%	0,9%	1,3%	0,1%	0,8%	0,0%	0,7%
Minimum			Minimum	3,5%	0,3%	0,4%	0,3%	2,7%	7,5%	0,4%
Maximum			Maximum	17,5%	1,6%	3,9%	0,5%	3,9%	7,5%	1,4%

**Howell, 1993** (63)	Oceania	154	13-17	7,0%		1,0%		5,0%		
Weighted Mean				7,0%		1,0%		5,0%		
Weighted Standard Deviation				0,0%		0,0%		0,0%		
Minimum				7,0%		1,0%		5,0%		
Maximum				7,0%		1,0%		5,0%		

Legend additional table S8: Prevalence of tooth anomalies: hypodontia, hyperdontia, hypo-hyperdontia, impacted/retained teeth, ectopic eruption, transposition according to geographical location is given in percentages, along with information on the continent, the number of subjects and the age range. Y : age range is noted, but if not available then mean +/- SD are noted, but * if SD not mentioned in article. . Y : age range is noted, but if not available then mean +/- SD are noted, but * if SD not mentioned in article **Abbreviations**: Y: years.

Additional table S9: Prevalence of space anomalies according to geographical location.

Authors	Continent		Age in Y	Crowding maxillary arch	Crowding mandibular arch	Crowding	Spacing maxillary arch	Spacing mandibular arch	Spacing	Midline diastema
**Dacosta, 1999** (41)	Africa	1028	11-18	21,7%	36,3%		45,9%	30,0%		
**Hassanali, 1993** (62)	Africa	412	3-16						44,4%	
**Kabue, 1995** (69)	Africa	221	3-6	4,0%	10,0%					
**Kolawole, 2019** (73)	Africa	992	1-12			21,7%			29,9%	
**Mtaya, 2009** (85)	Africa	1601	12-14			14,1%			21,9%	
**Mtaya, 2017** (86)	Africa	253	3-5			0,8%			19,8%	
**Muyasa, 2012** (88)	Africa	1382	12-15	38,6%	31,1%	47,2%			46,6%	
**Ng'ang'a, 1991** (89)	Africa	251	13-15	25,4%						
**Ng'ang'a, 1996** (90)	Africa	919	13-15	21,0%	18,0%		17,0%	13,0%		
**Onyeaso, 2004** (95)	Africa	636	12-17	5,2%	7,7%	7,2%				36,8%
**Rwakatema, 2007** (106)	Africa	289	12-15			41,2%			28,4%	
Weighted Mean				23,8%	24,8%	24,5%	32,2%	22,0%	32,6%	36,8%
Weighted Standard Deviation				11,8%	10,6%	15,9%	14,4%	8,5%	10,7%	0,0%
Minimum				4,0%	7,7%	0,8%	17,0%	13,0%	19,8%	36,8%
Maximum				38,6%	36,3%	47,2%	45,9%	30,0%	46,6%	36,8%
**Calzada Bandomo, 2014** (34)	America	210	5 – 11			9,3%				
**Campos-Arias, 2013** (35)	America	88	7.0*			48,9%				
**de Almeida, 2008** (43)	America	344	3.94*	7,0%	11,3%					
**de Araújo Guimarães, 2018** (44)	America	390	8-10	19,7%	10,5%				44,9%	3,6%
**de Muniz, 1986** (45)	America	1554	12-13	6,2%	6,7%		1,8%	1,3%		
**Frazao, 2006** (53)	America	13801	12 & 18	18,9%	12,9%	38,0%			21,4%	
**Gois, 2012** (55)	America	212	8-11			34,9%				
**Mail, 2015** (80)	America	50	12		48,0%				36,0%	28,0%
**Martins, 2009** (81)	America	264	10-12	6,1%	16,3%	40,1%				14,8%
**Martins, 2019** (82)	America	1612	11-14			51,9%			32,1%	23,7%

**Sánchez-Pérez, 2013** (108)	America	249	15			50,0%			7,2%	
**Thilander, 2001** (124)	America	4724	5-17			52,1%			25,9%	7,0%
Weighted Mean				17,3%	12,3%	42,1%	1,8%	1,3%	23,5%	11,1%
Weighted Standard Deviation				4,3%	2,7%	7,3%	0,0%	0,0%	4,7%	7,3%
Minimum				6,1%	6,7%	9,3%	1,8%	1,3%	7,2%	3,6%
Maximum				19,7%	48,0%	52,1%	1,8%	1,3%	44,9%	28,0%
**Abu Alhaija, 2005** (12)	Asia	1003	13-15			50,4%				
**Abumelha, 2018** (13)	Asia	526	6-12			36,5%				
**Al-Emran, 1990** (17)	Asia	500	13.5-14.5	19,4%	23,4%		17,0%	8,0%		3,6%
**Alkilzy, 2007** (18)	Asia	234	2-16							
**Araki, 2017** (22)	Asia	420	10-16			11,9%				
**Baskaradoss, 2013** (27)	Asia	300	11-15			41,2%			12,4%	7,4%
**Bhardwaj, 2011** (30)	Asia	622	16-17			62,4%			29,6%	
**Bhayya, 2011** (31)	Asia	1000	4 – 6	1,7%	4,6%					
**Bilgic, 2015** (32)	Asia	2329	12-16			65,2%			1,8%	
**Chauhan, 2013** (37)	Asia	1188	9-12			17,8%			1,5%	
**Esa, 2001** (48)	Asia	1519	12-13	40,6%	22,2%				17,4%	
**Gudipaneni, 2018** (58)	Asia	500	7-12			47,2%			27,2%	
**Jamilian, 2010** (65)	Asia	350	14-17			93,4%				
**Komazaki, 2012** (74)	Asia	963	12-15	67,4%	67,9%					2,9%
**Madiraju, 2021** (79)	Asia	282	8-9			39,7%			7,1%	
**Mohamed, 2014** (84)	Asia	106	8-10	24,5%	57,5%		62,2%	23,6%		
**Murshid, 2010** (87)	Asia	1024	13-15	39,0%	58,0%					
**Nguyen, 2014** (92)	Asia	200	12 & 18			54,0%				
**Sanadhya, 2014** (107)	Asia	947	12-15			40,2%			27,1%	15,3%
**Singh, 2011** (114)	Asia	927	12			45,4%			10,0%	9,8%
**Sundareswaran, 2019** (120)	Asia	1554	13-15			66,6%			15,0%	
**Sunil, 2019** (121)	Asia	100	13-17			70,0%				6,0%
**Uematsu, 2012** (127)	Asia	2378	12-13 & 15-16			19,9%				
**Yu, 2019** (132)	Asia	2810	7-9			28,4%			9,5%	
**Zhou, 2017** (133)	Asia	2335	3-5			6,5%			44,8%	

Weighted Mean				35,3%	35,4%	40,4%	24,9%	10,7%	16,7%	8,3%
Weighted Standard Deviation				21,3%	23,7%	22,2%	17,2%	5,9%	14,3%	4,8%
Minimum				1,7%	4,6%	6,5%	17,0%	8,0%	1,5%	2,9%
Maximum				67,4%	67,9%	93,4%	62,2%	23,6%	44,8%	15,3%

**Ciuffolo, 2005** (38)	Europe	810	11-14			20,2%			5,6%	
**Dimberg, 2015** (46)	Europe	277	3 & 7 & 11.5			31,0%			9,4%	6,5%
**Ferro, 2016** (51)	Europe	380	14	17,0%	19,0%	30,0%				1,0%
**Ferro, 2016** (52)	Europe	1960	3-5							
**Gàbris, 2006** (54)	Europe	483	16-18						7,8%	
**Ingervall, 1975** (64)	Europe	200	8-16	14,0%	12,0%		21,0%	10,5%		
**Johannsdottir, 1997** (67)	Europe	396	6	77,9%	83,3%				42,5%	
**Kasparviciene, 2014** (71)	Europe	709	5-7						52,2%	
**Perillo, 2010** (98)	Europe	703	12.2*			45,9%			22,9%	
**Perinetti, 2008** (99)	Europe	1198	7-11	4,0%	23,2%	17,2%				
**Robke, 2007** (103)	Europe	434	2-6			36,9%				
**Seemann, 2011** (109)	Europe	2975	4 & 7.8*			30,3%				
**Sepp, 2017** (111)	Europe	392	7.1-10.4	18,9%	37,9%	49,7%	57,7%	15,3%		73,0%
**Sepp, 2019** (112)	Europe	390	4-5	0,0%	0,3%					34,9%
**Sonnesen, 1998** (116)	Europe	104	7-13			56,7%			13,5%	
**Stahl, 2003** (117)	Europe	8864	2-10			10,1%			1,2%	
**Steinmassl, 2017** (119)	Europe	157	8-10	22,3%	31,8%		38,9%	17,2%		
**Tausche, 2004** (123)	Europe	1975	6-8	12,0%	14,3%					
**Todor, 2019** (126)	Europe	960	7-14			47,5%			3,5%	
Weighted Mean				15,6%	23,3%	28,1%	44,0%	14,4%	7,2%	30,9%
Weighted Standard Deviation				19,0%	19,4%	11,2%	15,7%	2,5%	13,5%	29,0%
Minimum				0,0%	0,3%	10,1%	21,0%	10,5%	1,2%	1,0%
Maximum				77,9%	83,3%	56,7%	57,7%	17,2%	52,2%	73,0%

**Howell, 1993** (63)	Oceania	154	13-17			72,0%			17,5%	
**Johnson, 2000** (68)	Oceania	294	9.9 - 11. 3	6,0%		80,3%			59,5%	

Weighted Mean				6,0%		77,4%			45,1%	
Weighted Standard Deviation				0,0%		3,9%			20,0%	
Minimum				6,0%		72,0%			17,5%	
Maximum				6,0%		80,3%			59,5%	

Legend additional table S9: Prevalence of space anomalies: crowding, spacing and midline diastema according to geographical location in percentages, along with information on the continent, the number of subjects and the age range. Y: age range is noted, but if not available then mean +/- SD are noted, but * if SD not mentioned in article **Abbreviations**: Y: years.

Additional file 10: protocol systematic review

PROTOCOL SYSTEMATIC REVIEW

Methodology

I.PIO Questions
(Prevalence of orthodontic malocclusions and problems)

PIO : What is the prevalence of the different orthodontic malocclusions and/or dental anomalies and/or maxillofacial syndromes and/or congenital abnormalities in children and adolescents?

Patient :	Children and adolescents (under the age of 18 years).
Intervention :	Assessment of malocclusion and/or dental characteristics or features.
Outcome :	Prevalence and/or incidence of dental malocclusion and/or dental anomalies and/or maxillofacial syndromes and/or congenital abnormalities

II.Determination of the search terms for the different research questions.

II.1. FOR PUBMED

Following Mesh terms and free terms are used to perform the search in PubMed:

((((("Orthodontics"[MeSH] OR Orthodont*[TIAB] OR Orthodontia*[TIAB] OR "Orthodontic care"[TIAB] OR "Orthodontic model"[TIAB] OR Orthodontology[TIAB] OR Orthodontolog*[TIAB]))) AND (("Infant"[MeSH] OR Infant*[TIAB] OR Newborn*[TIAB] OR Neonat*[TIAB] OR "Child"[MeSH] OR Child*[TIAB] OR "Adolescent"[MeSH] OR Adolescen*[TIAB] OR Teen*[TIAB] OR Youth[TIAB] OR "Minors"[MeSH] OR Minor*[TIAB] OR Baby*[TIAB] OR Newborn*[TIAB] OR "Preschool child"[TIAB] OR Juvenile*[TIAB] OR Schoolchild*[TIAB] OR Schoolgirl*[TIAB] OR Schoolboy*[TIAB] OR "School girl"[TIAB] OR "School boy"[TIAB] OR Teenage*[TIAB] OR Infanc*[TIAB] OR Childhood*[TIAB] OR Pubert*[TIAB]))) AND (("Malocclusion"[MeSH] OR Malocclusion*[TIAB] OR "Angle's Classification"[TIAB] OR "Dental malocclusion"[TIAB] OR "Dental malocclusions"[TIAB] OR "Cross Bite"[TIAB] OR "Cross Bites"[TIAB] OR Crossbite*[TIAB] OR "Forced Bite"[TIAB] OR "Tooth Crowding"[TIAB] OR "Malocclusion, Angle Class I"[MeSH] OR "Angle Class I malocclusion"[TIAB] OR "Angle Class I"[TIAB] OR "Malocclusion, Angle Class II"[MeSH] OR "Angle class II malocclusion"[TIAB] OR "Angle Class II"[TIAB] OR "Angle Class II Division 1"[TIAB] OR "Angle Class II Division 2"[TIAB] OR "Overbite"[MeSH] OR Overbite*[TIAB] OR "Malocclusion, Angle Class III"[MeSH] OR "Angle Class III malocclusion"[TIAB] OR "Angle Class III"[TIAB] OR Prognath*[TIAB] OR "Maxillary Discrepancy"[TIAB] OR "Maxillary Discrepancies"[TIAB] OR "Open bite"[TIAB] OR "Open bites"[TIAB] OR "Mandible protrusion"[TIAB] OR "Mandibular protrusion"[TIAB] OR Progenia*[TIAB] OR Micrognath*[TIAB] OR Retrognath*[TIAB] OR "Mandibular Retrusion"[TIAB] OR "Mandible Retrusion"[TIAB] OR "Maxillary Retrusion"[TIAB] OR "Mandibular hypoplasia"[TIAB] OR

"Maxillary hypoplasia"[TIAB] OR "Jaw abnormality"[TIAB] OR "Jaw abnormalities"[TIAB] OR "Jaw anomalies"[TIAB] OR "Jaw deformity"[TIAB] OR "Jaw deformities"[TIAB] OR "Jaw malformation"[TIAB] OR "Cleft lip"[MeSH] OR "Cleft lip"[TIAB] OR Harelip*[TIAB] OR "Cleft Palate"[MeSH] OR "Cleft Palate"[TIAB] OR "Cleft lip nose"[TIAB] OR "cleft lip palate"[TIAB] OR "Unilateral cleft lip"[TIAB] OR "Bilateral cleft lip"[TIAB] OR "Cheilognathopalatoschisis"[TIAB] OR "Cheilognatopalatoschizis"[TIAB] OR "Cheilopalatoschisis"[TIAB] OR "Palate malformation"[TIAB] OR "Palate malformation"[TIAB] OR "Tooth, Supernumerary"[MeSH] OR "Supernumerary tooth"[TIAB] OR "Supernumerary teeth"[TIAB] OR "Diastema"[MeSH] OR Diastema*[TIAB] OR "Fused Teeth"[MeSH] OR "Fused Teeth"[TIAB] OR "Fused Tooth"[TIAB] AND "Tooth Crowding"[TIAB] OR "Anodontia"[MeSH] OR Anodont*[TIAB] OR Agenes*[TIAB] OR Hypodont*[TIAB] OR Oligodont*[TIAB] OR "Double Tooth"[TIAB] OR "Fused Mandibular Incisor"[TIAB] OR "Tooth Ankylosis"[MESH] OR "Tooth Ankylosis"[TIAB] OR "Dentoalveolar Ankylosis"[TIAB] OR "Dental Ankylosis"[TIAB] OR "Teeth Ankylosis"[TIAB] OR "Tooth Eruption"[MeSH] OR "Tooth Eruption"[TIAB] OR "Posterior Open bite "[TIAB] OR "Tooth Eruption, Ectopic"[MeSH] OR "Ectopic Tooth Eruption"[TIAB] OR "Ectopic teeth"[TIAB] OR "Ectopic tooth"[TIAB] OR "Tooth, Impacted"[MeSH] OR "Impacted Tooth"[TIAB] OR "Impacted Teeth"[TIAB] OR "Impacted Molar"[TIAB] OR "Impacted Molars"[TIAB] OR "Impacted Canine"[TIAB] OR "Impacted Canines"[TIAB] OR "Tooth, Unerupted"[MeSH] OR "Unerupted Tooth"[TIAB] OR "Unerupted Teeth"[TIAB] OR "Serial extraction"[TIAB] OR "Serial extractions"[TIAB]))) AND (("Epidemiology"[MeSH] OR Epidemiolog*[TIAB] OR "Epidemiologic Studies"[MeSH] OR "Epidemiologic Study"[TIAB] OR "Epidemiologic Studies"[TIAB] OR "Epidemiological Study"[TIAB] OR "Epidemiological studies"[TIAB] OR "Epidemiological study design"[TIAB] OR "Epidemiologic research design"[MeSH] OR "Epidemiologic Methods"[MeSH] OR "Epidemiologic Methods"[TIAB] OR "Clinical epidemiology"[TIAB] OR "Epidemiologic factor"[TIAB] OR "Epidemiologic method"[TIAB] OR "Epidemiologic research"[TIAB] OR "Epidemiologic study design"[TIAB] OR "Epidemiologic characteristics"[TIAB] OR "Epidemiologic survey"[TIAB] OR "Epidemiological research"[TIAB] OR "Epidemiological research design"[TIAB] OR "Epidemiologic model"[TIAB] OR "Incidence"[MeSH] OR "Incidence"[TIAB] OR "Incidence study"[TIAB] OR "Incidence studies"[TIAB] OR "Incidence rate"[TIAB] OR "Occurrence"[TIAB] OR "Prevalence"[MeSH] OR "Prevalence"[TIAB] OR "Prevalence study"[TIAB] OR "Prevalence studies"[TIAB] OR "Frequency"[TIAB] OR "Occurence"[TIAB]))

II.2. FOR COCHRANE

Following Mesh terms and free terms are used to perform the search in Cochrane:

“Orthodontics” in Title Abstract Keyword AND “Infant “ OR “Child” OR “Adolescent” OR “Adolescen*” OR “Minors” in Title Abstract Keyword AND “Malocclusion” OR “Malocclusion, Angle Class I” OR “Malocclusion, Angle Class II” OR “Overbite”OR “Malocclusion, Angle Class III” OR “Jaw Abnormalities” OR “Craniofacial Abnormalities” OR “Maxillofacial syndromes” OR “Marfan syndrome” OR “Cleft lip”OR “Cleft Palate“ OR “Tooth Abnormalities” OR “Odontoma” OR “Tooth Ankylosis” OR “Tooth Eruption” OR “Tooth Eruption, Ectopic” OR “Tooth,Impacted” OR “Tooth, Unerupted” OR “Mesial Movement of Teeth” OR “Tooth Abrasion” OR “Tooth Erosion” OR “Tooth Extraction” OR “Tooth Fractures” OR “Cracked tooth syndrome” OR “ Tooth Germ” OR “Tooth Avulsion” OR “ Tooth Loss” OR “Tooth Migration” OR “Tooth Mobility” OR “Tooth Replantation” OR “Tooth Resorption” OR “Tooth Injuries” OR “Tooth Demineralization” OR “Dental Enamel Hypoplasia” OR “Tooth Attrition” OR “Dental Caries” in Title Abstract Keyword AND “Epidemiology” OR “Epidemiologic Studies” OR “Epidemiologic research design” OR “Epidemiologic Methods” OR “Incidence” OR “Incidence studies” OR “Prevalence” OR “Prevalence studies” in Title Abstract

II.3. FOR EMBASE

Following search terms were defined in analogy to PubMed search to perform the search in Embase, but by using the Emtree tools:

'orthodontics'/exp OR 'orthodontics' OR 'orthodont*':ti,ab OR 'orthodontia*':ti,ab OR 'orthodontic care*':ti,ab OR 'orthodontic model*':ti,ab OR 'orthodontology*':ti,ab

'infant'/exp OR 'infant' OR 'infant*':ti,ab OR 'baby*':ti,ab OR 'newborn*':ti,ab OR 'neonat*':ti,ab OR 'preschool child*':ti,ab OR 'juvenile'/exp OR 'juvenile' OR 'juvenile*':ti,ab OR 'youth*':ti,ab OR 'child'/exp OR 'child' OR 'child*':ti,ab OR 'school child*':ti,ab OR 'schoolgirl*':ti,ab OR 'adolescent'/exp OR 'adolescent' OR 'adolescent*':ti,ab OR 'teenager*':ti,ab OR 'teen*':ti,ab OR 'infancy'/exp OR 'infancy' OR 'infancy*':ti,ab OR 'childhood'/exp OR 'childhood' OR 'childhood*':ti,ab OR 'adolescence'/exp OR 'adolescence' OR 'adloscenc*':ti,ab OR 'teenage*':ti,ab OR 'puberty'/exp OR 'puberty' OR 'pubert*':ti,ab OR 'minor (person)'/exp OR 'minor (person)' OR 'minor*(person)':ti,ab

'malocclusion'/exp OR 'malocclusion' OR 'malocclusion*':ti,ab OR 'angle classification':ti,ab OR 'angle classes malocclusion':ti,ab OR 'dental malocclusion':ti,ab OR 'jaw malocclusion':ti,ab OR 'malocclusion angle class i':ti,ab OR 'angle class i':ti,ab OR 'malocclusion angle class ii':ti,ab OR 'angle class ii':ti,ab OR 'angle class ii, division 1':ti,ab OR 'angle class ii, division 2':ti,ab OR 'malocclusion angle class iii':ti,ab OR 'angle class iii':ti,ab OR 'mandibular prognathism':ti,ab OR 'maxillary discrepancy':ti,ab OR 'occlusion disorder, jaw':ti,ab OR 'jaw occlusion disorder':ti,ab OR 'occlusion,mal':ti,ab OR 'open bite':ti,ab OR 'overbite*':ti,ab OR 'prognathia'/exp OR 'prognathia' OR 'lower jaw prognathia':ti,ab OR 'lower jaw prognathism':ti,ab OR 'mandible prognathism':ti,ab OR 'mandibular prognathia':ti,ab OR 'mandible protrusion':ti,ab OR 'mandibular protrusion':ti,ab OR 'progenia*':ti,ab OR 'prognath*':ti,ab OR 'retrognathia'/exp OR 'retrognathia' OR 'retrognath*':ti,ab OR 'mandibular retroposition':ti,ab OR 'mandibular retrusion':ti,ab OR 'maxillary retroposition':ti,ab OR 'maxillary retrusion':ti,ab OR 'craniofacial abnormality':ti,ab OR 'craniofacial abnormalities':ti,ab OR 'receding jaw':ti,ab OR 'crossbite'/exp OR 'crossbite' OR 'crossbite':ti,ab OR 'cross bite':ti,ab OR 'jaw malformation'/exp OR 'jaw malformation' OR 'jaw malformation':ti,ab OR 'mandibular hypoplasy':ti,ab OR 'mandibular hypoplasia':ti,ab OR 'maxillary hypoplasy':ti,ab OR 'maxillary hypoplasia':ti,ab OR 'chin malformation':ti,ab OR 'jaw dismorfity':ti,ab OR 'jaw deformity':ti,ab OR 'jaw dismorfities':ti,ab OR 'jaw demorfities':ti,ab OR 'malformation chin':ti,ab OR 'malformation jaw':ti,ab OR 'mandible deformity':ti,ab OR 'mandible condyle agenesis':ti,ab OR 'mandibular defect':ti,ab OR 'mandibular malformation':ti,ab OR 'lip malformation'/exp OR 'lip malformation' OR 'lip malformation':ti,ab OR 'cleft lip':ti,ab OR 'bilateral cleft lip':ti,ab OR 'cleft lip palate':ti,ab OR 'cheilo gnathopalatoschisis':ti,ab OR 'cheilognathopalathoschisis':ti,ab OR 'cheilognathopalatoschisis':ti,ab OR 'cheilognatopalatoschizis':ti,ab OR 'cheilopalatoschisis':ti,ab OR 'cleft lip maxillo palate':ti,ab OR 'lip jaw palate cleft':ti,ab OR 'cleft lip nose'/exp OR 'cleft lip nose' OR 'cleft lip nose':ti,ab OR 'cleft lip face palate'/exp OR 'cleft lip face palate' OR 'cleft palate lift':ti,ab OR 'labiopalatoschis*':ti,ab OR 'palatolabioschis*':ti,ab OR 'unilateral cleft lip'/exp OR 'unilateral cleft lip' OR 'unilateral cleft lip':ti,ab OR 'cleft lip, unilateral':ti,ab OR 'palate malformation'/exp OR 'palate malformation' OR 'palate malformation':ti,ab OR 'cleft lip face palate':ti,ab OR 'cleft palate':ti,ab OR 'palate deformity':ti,ab OR 'palate deformities':ti,ab OR 'tooth malformation'/exp OR 'tooth malformation' OR 'tooth malformation':ti,ab OR 'tooth abnormalities':ti,ab OR 'tooth abnormality':ti,ab OR 'teeth abnormalities':ti,ab OR 'oligodonti*':ti,ab OR 'diastema*':ti,ab OR 'double tooth':ti,ab OR 'fused mandibular incisor':ti,ab OR 'tooth ankyloses':ti,ab OR 'dentoalveolar ankylosis':ti,ab OR 'dental ankylosis':ti,ab OR 'teeth ankyloses':ti,ab OR 'tooth eruption*':ti,ab OR 'failure of tooth eruption, primary*':ti,ab OR 'posterior openbite, familial':ti,ab OR 'primary retention of teeth':ti,ab OR 'primary failure of eruption, nonsyndromic':ti,ab OR 'dental noneruption':ti,ab OR 'ectopic tooth eruption':ti,ab OR 'eruption, ectopic tooth':ti,ab OR 'eruptions, ectopic tooth':ti,ab OR 'tooth eruptions, ectopic':ti,ab OR 'teeth, impacted':ti,ab OR 'tooth, unerupted':ti,ab OR 'teeth, unerupted':ti,ab OR 'premature extraction deciduous tooth':ti,ab OR 'premature extraction deciduous teeth':ti,ab OR 'premature extraction primary tooth':ti,ab OR 'primary extraction primary teeth':ti,ab OR 'serial extraction'/exp OR 'serial extraction' OR 'serial extraction':ti,ab OR 'extraction, serial':ti,ab OR 'extractions, serial':ti,ab OR 'premature loss tooth':ti,ab OR 'premature loss deciduous tooth':ti,ab OR 'premature loss teeth':ti,ab OR 'premature loss deciduous teeth':ti,ab OR 'premature loss primary tooth':ti,ab OR 'premature loss primary teeth':ti,ab OR 'fused teeth':ti,ab OR 'fused tooth':ti,ab OR 'supernumerary tooth':ti,ab OR

'supernumerary teeth':ti,ab OR 'anodont*':ti,ab OR 'crowding*':ti,ab OR 'hypodont*':ti,ab OR 'tooth injury':ti,ab OR 'tooth injuries':ti,ab OR 'dental disorder':ti,ab OR 'ectopic teeth':ti,ab OR 'ectopic tooth':ti,ab OR 'impacted molar':ti,ab OR 'impacted tooth':ti,ab OR 'impacted teeth':ti,ab OR 'tooth, impacted':ti,ab OR 'tooth eruption':ti,ab OR 'tooth eruption, ectopic':ti,ab OR 'tooth ankylosis':ti,ab OR 'unerupted tooth'/exp OR 'unerupted tooth' OR 'unerupted tooth':ti,ab OR 'unerupted teeth':ti,ab

'epidemiology'/exp OR 'epidemiolog*':ti,ab OR 'clinical epidemiolog*':ti,ab OR 'epidemiologic factor*':ti,ab OR 'epidemiologic method*':ti,ab OR 'epidemiologic research*':ti,ab OR 'epidemiologic stud*':ti,ab OR 'epidemiologic study design*':ti,ab OR 'epidemiologic characteristic*':ti,ab OR 'epidemiologic survey*':ti,ab OR 'epidemiological research*':ti,ab OR 'epidemiologic model*':ti,ab OR 'epidemiological stud*':ti,ab OR 'epidemiological study design*':ti,ab OR 'incidence'/exp OR 'incidence*':ti,ab OR 'incidence stud*':ti,ab OR 'occurrence*':ti,ab OR 'incidence rate*':ti,ab OR 'prevalence*':ti,ab OR 'prevalence stud*':ti,ab OR 'frequenc*':ti,ab OR 'occurenc*':ti,ab

II.4. FOR WEB OF SCIENCE

Following terms were used to perform the seach in Web of Science:

(Orthodontics OR Orthodont* OR Orthodontia* OR Orthodontic care* OR Orthodontic model* OR Orthodontology*) *AND* (Infant OR Infant* OR Newborn* OR Neonat*: OR Child OR Child* OR Adolescent OR Adolescen* OR Teen* OR Youth OR Minors OR Minor* OR baby* OR high risk infant* OR hospitalized infant* OR newborn* OR preschool child* OR juvenile* OR schoolchild* OR schoolgirl* OR schoolboy* OR school girl* OR school boy* OR teenage* OR infancy* OR childhood* OR pubert*Infant OR Infant* OR Newborn* OR Neonat*: OR Child OR Child* OR Adolescent OR Adolescen* OR Teen* OR Youth OR Minors OR Minor* OR baby* OR high risk infant* OR hospitalized infant* OR newborn* OR preschool child* OR juvenile* OR schoolchild* OR schoolgirl* OR schoolboy* OR school girl* OR school boy* OR teenage* OR infancy* OR childhood* OR pubert*) *AND* (Malocclusion OR Malocclusion* OR Angles Classification* OR Angles classes malocclusion OR dental malocclusion* OR jaw malocclusion* OR jaw occlusion disorder* OR Cross Bite* OR Crossbite OR Forced Bite* OR Tooth Crowding OR Malocclusion, Angle Class I OR Malocclusion, Angle Class I OR Angle Class I OR Malocclusion, Angle Class II OR Angle Class II OR Angle Class II, Division 1 OR Angle Class II, Division 2 OR Overbite OR Overbite* OR Malocclusion, Angle Class III OR Angle Class III OR Habsburg Jaw* OR Hapsburg Jaw * OR Prognathism, Mandibular* OR Prognath* OR Underbite* OR Maxillary Discrepanc* OR occlusion disorder, jaw* OR occlusion,mal* OR open bite* OR lower jaw prognath* OR mandible prognath* OR mandible protrusion* OR mandibula prognath* OR mandibular prognath* OR mandibular protrusion* OR progenia* Jaw Abnormalities OR Jaw Abnormalit* OR Cleft Palate OR Cleft lip* OR Cleft Nose OR Micrognath* OR Pierre Robin Syndrome* OR Retrognath* OR Mandibular Retroposition* OR Mandibular Retrusion* OR Maxillary Retroposition* OR Maxillary Retrusion* OR Craniofacial Abnormalities OR Craniofacial Abnormalit* OR Abnormalities, Craniofacial* OR Abnormality, Craniofacial* OR Maxillofacial syndromes OR Maxillofacial syndrome* OR Marfan syndrome OR Marfan syndrome* OR receding jaw* OR Jaw malformation* OR Goldenhar syndrome* OR hemifacial macrosom* OR mandible hypoplas* OR mandibulofacial dysostos* OR maxilla hypoplas* OR micrognath* OR Nager acrofacial dysostos* OR Pierre Robin syndrome* OR chin malformation* OR dysostosis mandibul* OR jaw abnormal* OR jaw anomal* OR jaw dismorfit* OR jaw deformit* OR jaw dismorfit* OR malformation,chin* OR malformation jaw* OR mandible deformit* OR mandible condyle agenes* OR mandibular defect* OR mandibular dysostos* OR mandibular malformat* OR Lip malformation* OR Cleft lip OR Cleft lip* OR Lip, Cleft* OR Lips, Cleft* OR Harelip* OR Cleft Palate OR Cleft Palate* OR Palate Cleft* OR Palates, Cleft* OR cleft lip, face, palate* OR cleft lip, nose* OR cleft lip palate* OR long philtrum* OR unilateral cleft lip* OR bilateral cleft lip* OR Van der Woude

syndrome* cleft lip palate* OR cheilo gnathopalatoschis* OR cheilognathopalathoschis* OR cheilognathopalatoschis* OR cheilognatopalatoschiz* OR cheilognathouranoschis* OR cheilognatopalatoschis* OR cheilopalatoschis* OR cleft lip maxillo palate* OR harelip* OR lip jaw palate cleft* OR cleft lip face palate* OR cleft lip nose* OR cleft lip face palate* OR cleft palate lift* OR labiopalatoschis* OR palatolabioschis* OR unilateral cleft lip* OR cleft lip, unilateral* OR lip, unilateral cleft* OR Palate malformation* OR palate malformation* OR cleft lip face palate* OR cleft palate* OR high arched palate* OR Roberts syndrome* OR velocardiofacial syndrome OR CLP OR CP OR UCLP OR Tooth Abnormalities OR Tooth Abnormalit* OR Teeth Abnormalit* OR tooth disease* Tooth Malformation* OR acro-dermato-ugual-lacrimal-tooth syndrome* OR amalogenesis imperfecta* OR dens evaginatus* OR enamel hypoplasia* OR invaginated tooth* invaginated tooth*OR oculodentodigital syndrome* OR Odontome* OR Tooth Supernumerary* OR Tooth Crowding* OR Anodontia* OR Agenes* OR Hypodontia* OR Oligodontia* OR Dens in dent* OR Dental Enamel Hypoplasia* OR Molar Incisor Hypomineralization* OR Amelogenesis Imperfecta* OR Congenital Enamel Hypoplas* OR Dentin Dysplasia* OR Hypoplastic Enamel* OR Hypoplasia Dental Enamel* OR Enamel Agenes* OR Enamel Hypoplas* OR Dentinogenesis Imperfecta* OR Diastema* OR Fused Teeth* OR Double Tooth* OR Fused Mandibular Incisor* OR Tooth, Supernumerary* OR Fourth Molar* OR Odontodysplasia* OR Ghost Teeth* OR Ghost Tooth* OR Odontogenic Dypslasia* OR Odontogenesis Imperfecta* OR Odontoma OR Odontom* OR Tooth Ankylosis OR Tooth Ankylos* OR Dentoalveolar Ankylos* OR Dental Ankylos* OR Teeth Ankylos* OR Tooth Eruption OR Tooth Eruption* OR Teething* OR Failure of Tooth Eruption, Primary* OR Posterior Openbite, Familial* OR Primary Retention of Teeth* OR Unerupted Second Primary Molar* OR Primary Failure of Eruption, Nonsyndromic* OR Dental Noneruption* OR Tooth Eruption, Ectopic OR Tooth Eruption, Ectopic* OR Ectopic Tooth Eruption* OR Eruption, Ectopic Tooth* OR Eruptions, Ectopic Tooth* OR Tooth Eruptions, Ectopic* OR Tooth,Impacted OR Tooth, Impacted* OR Impacted Tooth* OR Teeth, Impacted* OR Impacted Teeth* OR Tooth, Unerupted OR Tooth, Unerupted* OR Unerupted Tooth* OR Teeth, Unerupted* OR Unerupted Teeth* OR Mesial Movement of Teeth OR Mesial Movement of Teeth* OR Teeth Mesial Movement* OR Mesial Migration of Teeth* OR Teeth Mesial Migration* OR Mesial Drift of Teeth* OR Teeth Mesial Drift* OR Tooth Abrasion OR Tooth Abrasion* OR Abrasion, Tooth* OR Abrasion, Dental* OR Dental Abrasion* OR Tooth Erosion OR Tooth Erosion* OR Erosion, Tooth* OR Erosions, Tooth OR Tooth Extraction OR Tooth extraction* OR Extraction, Tooth* OR Extractions, Tooth* OR Premature extraction deciduous tooth* OR Premature extraction deciduous teeth* OR Premature extraction primary tooth* OR Primary extraction primary teeth* OR Serial extraction OR Serial extraction* OR Extraction, Serial* OR Extractions, Serial* OR Tooth Fractures OR Tooth Fracture* OR Fracture, Tooth* OR Fractures, Tooth* OR Cracked tooth syndrome OR Cracked tooth syndrome* OR Syndrome, Cracked Tooth* OR Syndromes, Cracked Tooth* OR Tooth Germ OR Tooth Germ* OR Germ, Tooth* OR Germs, Tooth* OR Tooth Avulsion OR Tooth Avulsion* OR Avulsion, Tooth* OR Avulsions, Tooth* OR Avulsed Tooth* OR Tooth, Avulsed* OR Dislocation, Tooth* OR Dislocations, Tooth* OR Tooth Dislocation* OR Tooth Luxation* OR Luxation, Tooth* OR Luxations, Tooth* OR Transposition tooth* OR Transposition teeth* OR Tooth, Transposition* OR Teeth, Transposition* OR Tooth Loss OR Tooth Loss* OR Loss, Tooth* OR Premature loss tooth* OR Premature loss deciduous tooth* OR Premature loss teeth* OR Premature loss deciduous teeth* OR Premature loss primary tooth* OR Premature loss primary teeth* OR Tooth Migration OR Migration, Tooth* OR Tooth Drifting* OR Tooth Drift* OR Tooth Mobility OR Tooth Mobilit* OR Mobilities, Tooth* OR Mobility, Tooth* OR Tooth Replantation OR Tooth Replantation* OR Replantation, Tooth* OR Replantations, Tooth* OR Reimplantation, Tooth* OR Reimplantations, Tooth* OR Tooth Reimplantation* OR Tooth Resorption OR Tooth Resorption* OR Resorption, Tooth* OR Resorptions, Tooth* OR Tooth Injuries OR Tooth Injur* OR Injuries, Teeth* OR Injury, Teeth* OR Teeth Injur* OR Injuries, Tooth* OR Injury, Tooth* OR Tooth Demineralization OR Tooth Demineralization* OR Tooth Hypomineralization* OR Hypomineralization, Tooth* OR Hypomineralizations, Tooth* OR Demineralization, Tooth* OR Dental Enamel Hypoplasia OR Dental Enamel Hypoplasia* OR Hypoplastic Enamel* OR Enamel, Hypoplastic* OR Enamel Hypoplasia, Dental* OR Hypoplasia, Dental Enamel* OR Enamel Agenesis* OR Ageneses, Enamel* OR Agenesis, Enamel* OR Enamel Ageneses* OR Enamel Hypoplas* OR Hypoplasia, Enamel* OR Hypoplasias, Enamel* OR Molar

Incisor Hypomineralization* OR Hypomineralization, Molar Incisor* OR Tooth Attrition OR Tooth Attrition* OR Attrition, Tooth* OR Dental Attrition* OR Occlusal Wear* OR Wear, Occlusal* OR Wears, Occlusal* OR Attrition, Dental* OR Dental Caries OR Dental Caries* OR Dental Decay* OR Caries, Dental* OR Decay, Dental* OR Carious Dentin* OR Dentin, Carious* OR Dentins, Carious* OR Dental White Spot* OR White Spots, Dental* OR White Spot* OR Spot, White* OR Spots, White* OR Dental White Spots* OR White Spot, Dental* OR dental abnomal* OR tooth anomal* OR tooth deformit* OR tooth dysplas* OR fused teeth* OR fused tooth* OR supernumerary tooth* OR supernumerary teeth*OR anodontia* OR crowding* OR dental caries* OR hypodont* OR tooth injur* OR dental disorder* OR ectopic teeth* OR ectopic tooth* OR impacted molar* OR impacted tooth* OR impacted teeth* OR tooth, impacted* OR tooth eruption* OR tooth eruption, ectopic* OR tooth luxation* OR tooth resorption* OR tooth root resorption* OR tooth wear* OR tooth ankylose* OR premature tooth loss* OR premature loss deciduous tooth* OR premature loss deciduous teeth* OR unerupted tooth* OR unerupted teeth*) *AND* (Epidemiology OR Epidemiolog* OR Epidemiologic Studies OR Epidemiologic Stud* OR Epidemiological Stud* OR epidemiological stud* OR epidemiological study design* OR Epidemiologic research design OR Epidemiologic research design* OR Epidemiologic Methods OR Epidemiologic Method* OR Epidemiol* OR clinical epidemiolog* OR cohort effect OR confounding factor* OR epidemiologic* OR epidemiologic factor* OR epidemiologic method* OR epidemiologic research* OR epidemiologic study design* OR epidemiologic characteristic* OR epidemiologic survey* OR epidemiological research* OR epidemiological research design* OR epidemiologic model* OR epidemiometr* OR Incidence OR Incidence* OR Incidence studies OR Incidence stud* OR incidence rate* OR Occurrence* OR Prevalence OR Prevalence* OR Prevalence studies OR Prevalence stud* OR prevalence* OR Frequenc* OR Occurenc*)

<u>II.5. FOR OPEN GREY</u>

Following terms were used to perform the seach in Open Grey:

Orthodontics OR Orthodont* OR orthodontia* OR orthodontic care*OR orthodontic model* OR orthodontology*

AND

Infant* OR Newborn* OR Neonat* OR Child* OR Adolescent OR Adolescen* OR Teen* OR Youth OR Minor* OR baby* OR high risk infant* OR hospitalized infant* OR newborn* OR preschool child* OR juvenile* OR schoolchild* OR schoolgirl* OR schoolboy* OR school girl* OR school boy* OR teenage* OR infancy* OR childhood* OR pubert*

AND

Malocclusion* OR Angle’s Classification* OR Cross Bite* OR Crossbite* OR Forced bite* OR Tooth Crowding* OR Maxillary Discrepancy* OR Angle’s Classification*OR Angle’s classes malocclusion* OR Malocclusion, Angle Class I*OR Angle Class I* OR Malocclusion, Angle Class II* OR Angle Class II* OR Angle Class II, Division 1* OR Angle Class II, Division 2*OR Class II Malocclusion, Division 1* OR Class II Malocclusion, Division 2* OR Malocclusion, Angle Class II, Division 1 *OR Malocclusion, Angle Class II, Division 2* OR Overbite*OR Malocclusion, Angle Class* OR Angle Class III* OR Habsburg Jaw* OR Hapsburg Jaw* OR Prognathism, Mandibular* OR Mandibular Prognathism*OR Prognathism* OR Underbite* OR Jaw Abnormalit* OR Jaw Abnormal* OR Cleft Palate* OR Cleft Lip and Palate* OR Cleft lip,Palate* OR Cleft Lip and Nose and Palate* OR Cleft Lip, Nose, Palate* OR Cleft Lip, Nose and Palate* OR Micrognathism * OR Pierre Robin Syndrome* OR Prognathism* OR Retrognathia* OR Mandibular Retroposition* OR Mandibular Retrusion* OR Maxillary Retroposition* OR Maxillary Retrusion* OR Retrognathism* OR Tooth Abnormalit* OR Teeth Abnormalit* OR Anodontia* OR Dens in Dente* OR Dental Enamel Hypoplasia* OR Amelogenesis Imperfecta* OR Dentin Dysplasia* OR Dentinogenesis Imperfecta* OR Diastema* OR Fused Teeth* OR Tooth,

Supernumerary* OR Odontodysplas* OR Tooth supernumerary* OR Odontom* OR Tooth Crowding* OR Tooth Ankylosis* OR Ankylosis of Teeth* OR Ankylosis, Dentoalveolar* OR Ankylosis, Tooth* OR Dental Ankylosis* OR Dentoalveolar Ankylosis* OR Tooth Eruption* OR Tooth Eruption,Ectopic* OR Tooth, Impacted*OR Teeth, Impacted* OR Tooth, Unerupted* OR Teeth, unerupted * OR Mesial Movement of Teeth* OR Tooth Abrasion* OR Tooth Discoloration* OR Tooth Erosion* OR Tooth Extraction* OR Premature extraction deciduous teeth and - tooth and - primary teeth and – tooth* OR Tooth Fractures* OR Tooth Germ* OR Tooth Avulsion*OR Avulsed Tooth*OR Dislocation, Tooth*OR Tooth Luxation* OR Tooth Loss* OR Tooth, premature loss* OR Tooth Migration* OR Tooth Drift* OR Tooth Mobility* OR Tooth Replantation* OR Tooth Resorption* OR Tooth Injuries* OR Injuries, Teeth* OR Injuries, Tooth* OR Teeth Injuries* OR Tooth, Deciduous* OR Tooth, Permanent* OR Tooth Demineralization* OR Dental Enamel Hypoplasia* OR Tooth Attrition* OR Dental Caries* OR Tooth Caries* OR Carious Dentin* OR Dental Decay* OR Dental White Spot* OR Dental White Spots* OR White Spots* OR Syndrome* OR Congenital Abnormalities* OR Congenital Abnormality* OR Birth Defects* OR Congenital Defects* OR Congenital Defect* OR Defomit* OR Congenital Disorder* OR OR Maxillofacial Syndrom* OR Marfan syndrome* OR Craniofacial Abnormal* OR Jaw malformation* OR Goldenhar syndrome* OR hemifacial microsomia* OR mandibular hypoplasia* OR mandibulofacial dysostosis* OR maxillar hypoplasia* OR micrognathia* OR Nager acrofacial dysostosis* OR Pierre Robin syndrome* OR Lip malformation* OR Cleft lip* OR Cleft Palate* OR Cleft Lip, Face, Palate* OR Cleft Lip Nose* OR Cleft Lip, Palate* OR Long Philtrum* OR Unilateral Cleft Lip* OR Van der Woude syndrome* OR Palate malformation* OR Cleft lip, face, palate* OR cleft palate* OR High arched palate* OR Roberts syndrome* OR Van de Woude syndrome* OR Velocardiofacial syndrome* OR Tooth malformation* OR acro-dermato-ugual-lacrimal-tooth syndrome* OR amalogenesis imperfecta* OR dens evaginatus* OR enamel hypoplasia* OR invaginated tooth* OR oculodentodigital syndrome* OR oligodontia* OR Van der Woude syndrome* AND

Epidemiologic Studies* OR Epidemiological Studies* OR Epidemiolog* Stud* OR Epidemiologic research design* OR Epidemiologic Research Designs* OR Epidemiological Research Design* OR Epidemiologic Methods* OR DMF Ind* OR Indexe, DMF* OR Epidemiology* OR Epidemiol* OR Frequenc* OR Incidence* OR Incidence studies* OR Occurrenc* OR Prevalence* OR Prevalence studies* OR Frequenc* OR Occurrenc*

III. Databases to be searched

**- PubMed*

**- Cochrane database of Systematic Research*

** - Embase*

**- Grey literature*

-Eagle (Europe) : opengrey.eu

**-Web of science*

IV. Inclusion and exclusion criteria

The following inclusion and exclusion criteria will be used for the evaluation of the titles and/or abstracts of the retrieved articles.

	**Inclusion criteria**	**Exclusion criteria**
**Terms**	All terms as described above	/
**Population**	Subjects under or equal to 18 Years old (children and adolescents)	Subjects older than 18 Years old
**Language**	English, Dutch, French, German, Spanish, and Portuguese	Other than the included articles.
**Study design**	Epidemiological Studies, RCT's, Systematic Reviews, Cochrane Reviews, Meta-analysis, Guidelines and Official Government Reports	Case Reports, Pilot Studies, Animal Studies, Editorials, Comment Articles, Conference proceedings, and Unpublished studies
**Topic**	Prevalence, incidence and epidemiological data of dental malocclusions and/or features and/or dento- and maxillofacial disorders/discrepancies/anomalies or dental malocclusions and/or features.	Articles concerning technical aspects orthodontic treatment, articles about orthodontic treatment methods.<br>Articles concerning treated patients or patients in treatment.

V. Collecting the studies

It is possible that studies in spite of the above described inclusion-exclusion-criteria some studies with a lower degree of evidence will need to be included.

VI. Application of the inclusion-exclusion criteria to the studies

Flowchart of included and excluded studies. (PRISMA DIAGRAM)

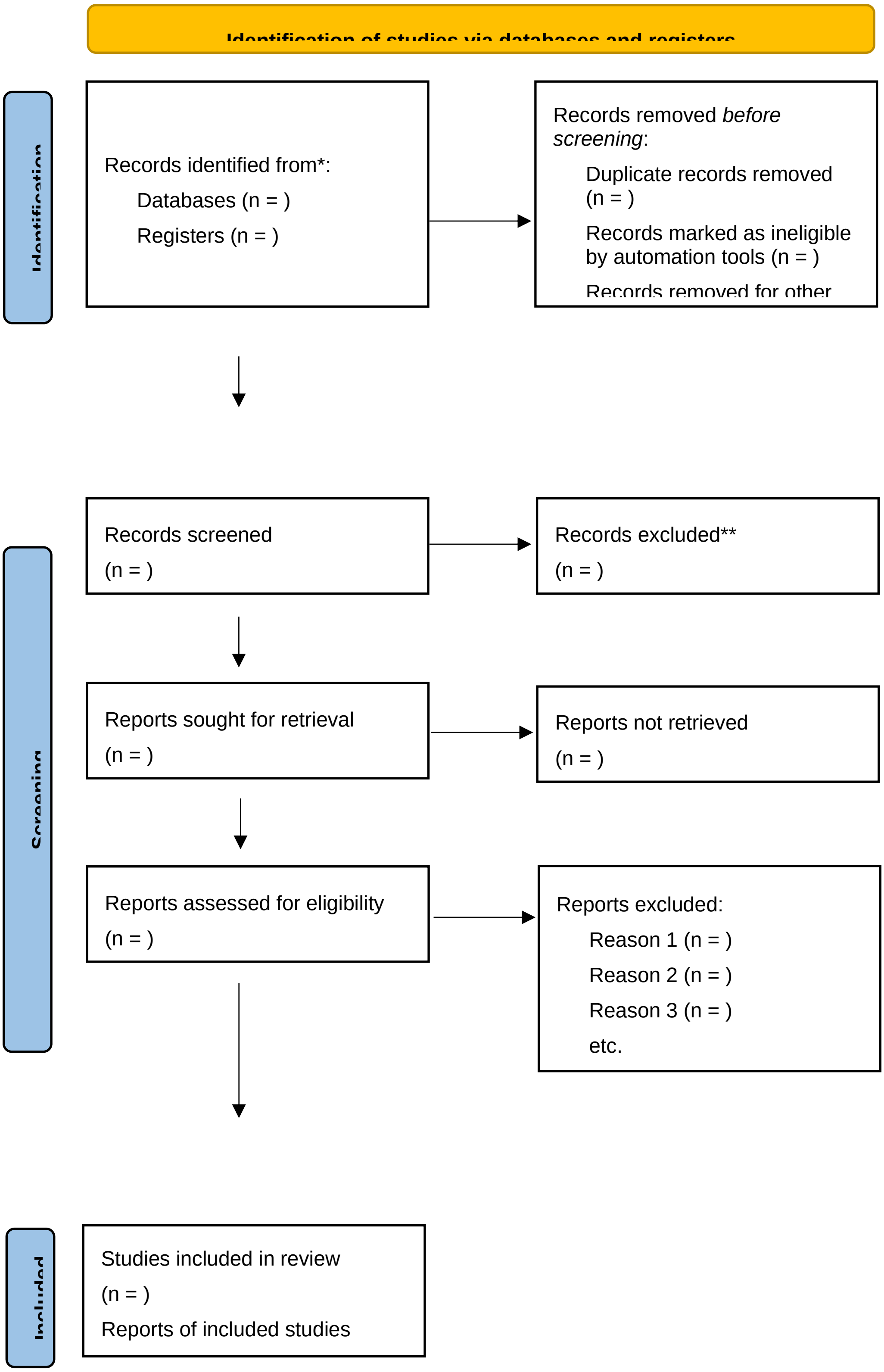

*Consider, if feasible to do so, reporting the number of records identified from each database or register searched (rather than the total number across all databases/registers).

**If automation tools were used, indicate how many records were excluded by a human and how many were excluded by automation tools.

From: Page MJ, McKenzie JE, Bossuyt PM, Boutron I, Hoffmann TC, Mulrow CD, et al. The PRISMA 2020 statement: an updated guideline for reporting systematic reviews. BMJ 2021;372:n71. doi: 10.1136/bmj.n71

For more information, visit: http://www.prisma-statement.org/

VII. Risk of Bias Assessment

The Methodological Index for Non-Randomized Studies (MINORS) from Slim et al., 2003, was used to assess the risk of bias of the included studies [10]. This tool contains 12 items related to comparative studies, the first 8 of which are also applied to non-comparative studies. Each item on the MINORS tool is scored as 0 (not reported), 1 (reported but inadequate), or 2 (reported and adequate), resulting in an ideal total score of 16 for non-comparative studies and 24 for comparative studies.

Slim, K.; Nini, E.; Forestier, D.; Kwiatkowski, F.; Panis, Y.; Chipponi, J. Methodological index for non-randomized studies (MINORS): Development and validation of a new instrument. *ANZ J. Surg.* **2003**, *73*, 712–716.

VIII. Collection of the data in tables.

According to Cochrane Checklists .

Study	Author, publication year
Methods	Study design
Participants	Number of participants, type of participant, country in which the study was performed, age and gender
Interventions	Clinical examination, X-ray analysis, Study Casts, Photographs, Interview/Questionnaire, Registration method.
Outcomes	Description and assessment of the prevalence of studied parameters (Angle Class I, Angle Class II, Angle Class II,1, Angle Class II,2, Angle Class III, overjet, reversed overjet, open bite, crowding, spacing, crossbite, scissor bite, forced bite (crossbite with lateral or frontal shift), hypodontia, supernumerary teeth, dental anomalies, impacted/retained teeth, ectopic teeth eruption, tooth transposition, and oral habits).
Notes	If applicable
Allocation concealment	Epidemiological data collected in a systematic way

Additional file S11: search strings

SEARCH STRINGS

Search string PubMed

((((("Orthodontics"[MeSH] OR Orthodont*[TIAB] OR Orthodontia*[TIAB] OR "Orthodontic care"[TIAB] OR "Orthodontic model"[TIAB] OR Orthodontology[TIAB] OR Orthodontolog*[TIAB]))) AND (("Infant"[MeSH] OR Infant*[TIAB] OR Newborn*[TIAB] OR Neonat*[TIAB] OR "Child"[MeSH] OR Child*[TIAB] OR "Adolescent"[MeSH] OR Adolescen*[TIAB] OR Teen*[TIAB] OR Youth[TIAB] OR "Minors"[MeSH] OR Minor*[TIAB] OR Baby*[TIAB] OR Newborn*[TIAB] OR "Preschool child"[TIAB] OR Juvenile*[TIAB] OR Schoolchild*[TIAB] OR Schoolgirl*[TIAB] OR Schoolboy*[TIAB] OR "School girl"[TIAB] OR "School boy"[TIAB] OR Teenage*[TIAB] OR Infanc*[TIAB] OR Childhood*[TIAB] OR Pubert*[TIAB]))) AND (("Malocclusion"[MeSH] OR Malocclusion*[TIAB] OR "Angle's Classification"[TIAB] OR "Dental malocclusion"[TIAB] OR "Dental malocclusions"[TIAB] OR "Cross Bite"[TIAB] OR "Cross Bites"[TIAB] OR Crossbite*[TIAB] OR "Forced Bite"[TIAB] OR "Tooth Crowding"[TIAB] OR "Malocclusion, Angle Class I"[MeSH] OR "Angle Class I malocclusion"[TIAB] OR "Angle Class I"[TIAB] OR "Malocclusion, Angle Class II"[MeSH] OR "Angle class II malocclusion"[TIAB] OR "Angle Class II"[TIAB] OR "Angle Class II Division 1"[TIAB] OR "Angle Class II Division 2"[TIAB] OR "Overbite"[MeSH] OR Overbite*[TIAB] OR "Malocclusion, Angle Class III"[MeSH] OR "Angle Class III malocclusion"[TIAB] OR "Angle Class III"[TIAB] OR Prognath*[TIAB] OR "Maxillary Discrepancy"[TIAB] OR "Maxillary Discrepancies"[TIAB] OR "Open bite"[TIAB] OR "Open bites"[TIAB] OR "Mandible protrusion"[TIAB] OR "Mandibular protrusion"[TIAB] OR Progenia*[TIAB] OR Micrognath*[TIAB] OR Retrognath*[TIAB] OR "Mandibular Retrusion"[TIAB] OR "Mandible Retrusion"[TIAB] OR "Maxillary Retrusion"[TIAB] OR "Mandibular hypoplasia"[TIAB] OR "Maxillary hypoplasia"[TIAB] OR "Jaw abnormality"[TIAB] OR "Jaw abnormalities"[TIAB] OR "Jaw anomalies"[TIAB] OR "Jaw deformity"[TIAB] OR "Jaw deformities"[TIAB] OR "Jaw malformation"[TIAB] OR "Cleft lip"[MeSH] OR "Cleft lip"[TIAB] OR Harelip*[TIAB] OR "Cleft Palate"[MeSH] OR "Cleft Palate"[TIAB] OR "Cleft lip nose"[TIAB] OR "cleft lip palate"[TIAB] OR "Unilateral cleft lip"[TIAB] OR "Bilateral cleft lip"[TIAB] OR "Cheilognathopalatoschisis"[TIAB] OR "Cheilognatopalatoschizis"[TIAB] OR "Cheilopalatoschisis"[TIAB] OR "Palate malformation"[TIAB] OR "Palate malformation"[TIAB] OR "Tooth, Supernumerary"[MeSH] OR "Supernumerary tooth"[TIAB] OR "Supernumerary teeth"[TIAB] OR "Diastema"[MeSH] OR Diastema*[TIAB] OR "Fused Teeth"[MeSH] OR "Fused Teeth"[TIAB] OR "Fused Tooth"[TIAB] AND "Tooth Crowding"[TIAB] OR "Anodontia"[MeSH] OR Anodont*[TIAB] OR Agenes*[TIAB] OR Hypodont*[TIAB] OR Oligodont*[TIAB] OR "Double Tooth"[TIAB] OR "Fused Mandibular Incisor"[TIAB] OR "Tooth Ankylosis"[MESH] OR "Tooth Ankylosis"[TIAB] OR "Dentoalveolar Ankylosis"[TIAB] OR "Dental Ankylosis"[TIAB] OR "Teeth Ankylosis"[TIAB] OR "Tooth Eruption"[MeSH] OR "Tooth Eruption"[TIAB] OR "Posterior Open bite "[TIAB] OR "Tooth Eruption, Ectopic"[MeSH] OR "Ectopic Tooth Eruption"[TIAB] OR "Ectopic teeth"[TIAB] OR "Ectopic tooth"[TIAB] OR "Tooth, Impacted"[MeSH] OR "Impacted Tooth"[TIAB] OR "Impacted Teeth"[TIAB] OR "Impacted Molar"[TIAB] OR "Impacted Molars"[TIAB] OR "Impacted Canine"[TIAB] OR "Impacted Canines"[TIAB] OR "Tooth, Unerupted"[MeSH] OR "Unerupted Tooth"[TIAB] OR "Unerupted Teeth"[TIAB] OR "Serial extraction"[TIAB] OR "Serial extractions"[TIAB]))) AND (("Epidemiology"[MeSH] OR Epidemiolog*[TIAB] OR "Epidemiologic Studies"[MeSH] OR "Epidemiologic Study"[TIAB] OR "Epidemiologic Studies"[TIAB] OR "Epidemiological Study"[TIAB] OR "Epidemiological studies"[TIAB] OR "Epidemiological study design"[TIAB] OR "Epidemiologic research design"[MeSH] OR "Epidemiologic Methods"[MeSH] OR "Epidemiologic Methods"[TIAB] OR "Clinical epidemiology"[TIAB] OR "Epidemiologic factor"[TIAB] OR "Epidemiologic method"[TIAB] OR "Epidemiologic research"[TIAB] OR "Epidemiologic study design"[TIAB] OR "Epidemiologic characteristics"[TIAB] OR "Epidemiologic survey"[TIAB] OR "Epidemiological research"[TIAB] OR "Epidemiological research design"[TIAB] OR "Epidemiologic model"[TIAB] OR "Incidence"[MeSH] OR "Incidence"[TIAB] OR "Incidence study"[TIAB] OR "Incidence studies"[TIAB] OR "Incidence rate"[TIAB] OR

"Occurrence"[TIAB] OR "Prevalence"[MeSH] OR "Prevalence"[TIAB] OR "Prevalence study"[TIAB] OR "Prevalence studies"[TIAB] OR "Frequency"[TIAB] OR "Occurence"[TIAB]))

Search string Cochrane

“Orthodontics” in Title Abstract Keyword AND “Infant “ OR “Child” OR “Adolescent” OR “Adolescen*” OR “Minors” in Title Abstract Keyword AND “Malocclusion” OR “Malocclusion, Angle Class I” OR “Malocclusion, Angle Class II” OR “Overbite”OR “Malocclusion, Angle Class III” OR “Jaw Abnormalities” OR “Craniofacial Abnormalities” OR “Maxillofacial syndromes” OR “Marfan syndrome” OR “Cleft lip”OR “Cleft Palate“ OR “Tooth Abnormalities” OR “Odontoma” OR “Tooth Ankylosis” OR “Tooth Eruption” OR “Tooth Eruption, Ectopic” OR “Tooth,Impacted” OR “Tooth, Unerupted” OR “Mesial Movement of Teeth” OR “Tooth Abrasion” OR “Tooth Erosion” OR “Tooth Extraction” OR “Tooth Fractures” OR “Cracked tooth syndrome” OR “ Tooth Germ” OR “Tooth Avulsion” OR “ Tooth Loss” OR “Tooth Migration” OR “Tooth Mobility” OR “Tooth Replantation” OR “Tooth Resorption” OR “Tooth Injuries” OR “Tooth Demineralization” OR “Dental Enamel Hypoplasia” OR “Tooth Attrition” OR “Dental Caries” in Title Abstract Keyword AND “Epidemiology” OR “Epidemiologic Studies” OR “Epidemiologic research design” OR “Epidemiologic Methods” OR “Incidence” OR “Incidence studies” OR “Prevalence” OR “Prevalence studies” in Title Abstract

Search string Embase

'orthodontics'/exp OR 'orthodontics' OR 'orthodont*':ti,ab OR 'orthodontia*':ti,ab OR 'orthodontic care*':ti,ab OR 'orthodontic model*':ti,ab OR 'orthodontology*':ti,ab

'infant'/exp OR 'infant' OR 'infant*':ti,ab OR 'baby*':ti,ab OR 'newborn*':ti,ab OR 'neonat*':ti,ab OR 'preschool child*':ti,ab OR 'juvenile'/exp OR 'juvenile' OR 'juvenile*':ti,ab OR 'youth*':ti,ab OR 'child'/exp OR 'child' OR 'child*':ti,ab OR 'school child*':ti,ab OR 'schoolgirl*':ti,ab OR 'adolescent'/exp OR 'adolescent' OR 'adolescent*':ti,ab OR 'teenager*':ti,ab OR 'teen*':ti,ab OR 'infancy'/exp OR 'infancy' OR 'infancy*':ti,ab OR 'childhood'/exp OR 'childhood' OR 'childhood*':ti,ab OR 'adolescence'/exp OR 'adolescence' OR 'adloscenc*':ti,ab OR 'teenage*':ti,ab OR 'puberty'/exp OR 'puberty' OR 'pubert*':ti,ab OR 'minor (person)'/exp OR 'minor (person)' OR 'minor*(person)':ti,ab

'malocclusion'/exp OR 'malocclusion' OR 'malocclusion*':ti,ab OR 'angle classification':ti,ab OR 'angle classes malocclusion':ti,ab OR 'dental malocclusion':ti,ab OR 'jaw malocclusion':ti,ab OR 'malocclusion angle class i':ti,ab OR 'angle class i':ti,ab OR 'malocclusion angle class ii':ti,ab OR 'angle class ii':ti,ab OR 'angle class ii, division 1':ti,ab OR 'angle class ii, division 2':ti,ab OR 'malocclusion angle class iii':ti,ab OR 'angle class iii':ti,ab OR 'mandibular prognathism':ti,ab OR 'maxillary discrepancy':ti,ab OR 'occlusion disorder, jaw':ti,ab OR 'jaw occlusion disorder':ti,ab OR 'occlusion,mal':ti,ab OR 'open bite':ti,ab OR 'overbite*':ti,ab OR 'prognathia'/exp OR 'prognathia' OR 'lower jaw prognathia':ti,ab OR 'lower jaw prognathism':ti,ab OR 'mandible prognathism':ti,ab OR 'mandibular prognathia':ti,ab OR 'mandible protrusion':ti,ab OR 'mandibular protrusion':ti,ab OR 'progenia*':ti,ab OR 'prognath*':ti,ab OR 'retrognathia'/exp OR 'retrognathia' OR 'retrognath*':ti,ab OR 'mandibular retroposition':ti,ab OR 'mandibular retrusion':ti,ab OR 'maxillary retroposition':ti,ab OR 'maxillary retrusion':ti,ab OR 'craniofacial abnormality':ti,ab OR 'craniofacial abnormalities':ti,ab OR 'receding jaw':ti,ab OR 'crossbite'/exp OR 'crossbite' OR 'crossbite':ti,ab OR 'cross bite':ti,ab OR 'jaw malformation'/exp OR 'jaw malformation' OR 'jaw malformation':ti,ab OR 'mandibular hypoplasy':ti,ab OR 'mandibular hypoplasia':ti,ab OR 'maxillary hypoplasy':ti,ab OR 'maxillary hypoplasia':ti,ab OR 'chin malformation':ti,ab OR 'jaw dismorfity':ti,ab OR 'jaw deformity':ti,ab OR 'jaw dismorfities':ti,ab OR 'jaw demorfities':ti,ab OR 'malformation chin':ti,ab OR 'malformation jaw':ti,ab OR 'mandible deformity':ti,ab OR 'mandible condyle agenesis':ti,ab OR 'mandibular defect':ti,ab OR 'mandibular malformation':ti,ab OR 'lip malformation'/exp OR 'lip malformation' OR 'lip malformation':ti,ab OR 'cleft lip':ti,ab OR 'bilateral cleft lip':ti,ab OR 'cleft lip palate':ti,ab OR 'cheilo gnathopalatoschisis':ti,ab OR 'cheilognathopalathoschisis':ti,ab OR 'cheilognathopalatoschisis':ti,ab OR 'cheilognatopalatoschizis':ti,ab OR 'cheilopalatoschisis':ti,ab OR 'cleft lip maxillo palate':ti,ab OR 'lip

jaw palate cleft':ti,ab OR 'cleft lip nose'/exp OR 'cleft lip nose' OR 'cleft lip nose':ti,ab OR 'cleft lip face palate'/exp OR 'cleft lip face palate' OR 'cleft palate lift':ti,ab OR 'labiopalatoschis*':ti,ab OR 'palatolabioschis*':ti,ab OR 'unilateral cleft lip'/exp OR 'unilateral cleft lip' OR 'unilateral cleft lip':ti,ab OR 'cleft lip, unilateral':ti,ab OR 'palate malformation'/exp OR 'palate malformation' OR 'palate malformation':ti,ab OR 'cleft lip face palate':ti,ab OR 'cleft palate':ti,ab OR 'palate deformity':ti,ab OR 'palate deformities':ti,ab OR 'tooth malformation'/exp OR 'tooth malformation' OR 'tooth malformation':ti,ab OR 'tooth abnormalities':ti,ab OR 'tooth abnormality':ti,ab OR 'teeth abnormalities':ti,ab OR 'oligodonti*':ti,ab OR 'diastema*':ti,ab OR 'double tooth':ti,ab OR 'fused mandibular incisor':ti,ab OR 'tooth ankyloses':ti,ab OR 'dentoalveolar ankylosis':ti,ab OR 'dental ankylosis':ti,ab OR 'teeth ankyloses':ti,ab OR 'tooth eruption*':ti,ab OR 'failure of tooth eruption, primary*':ti,ab OR 'posterior openbite, familial':ti,ab OR 'primary retention of teeth':ti,ab OR 'primary failure of eruption, nonsyndromic':ti,ab OR 'dental noneruption':ti,ab OR 'ectopic tooth eruption':ti,ab OR 'eruption, ectopic tooth':ti,ab OR 'eruptions, ectopic tooth':ti,ab OR 'tooth eruptions, ectopic':ti,ab OR 'teeth, impacted':ti,ab OR 'tooth, unerupted':ti,ab OR 'teeth, unerupted':ti,ab OR 'premature extraction deciduous tooth':ti,ab OR 'premature extraction deciduous teeth':ti,ab OR 'premature extraction primary tooth':ti,ab OR 'primary extraction primary teeth':ti,ab OR 'serial extraction'/exp OR 'serial extraction' OR 'serial extraction':ti,ab OR 'extraction, serial':ti,ab OR 'extractions, serial':ti,ab OR 'premature loss tooth':ti,ab OR 'premature loss deciduous tooth':ti,ab OR 'premature loss teeth':ti,ab OR 'premature loss deciduous teeth':ti,ab OR 'premature loss primary tooth':ti,ab OR 'premature loss primary teeth':ti,ab OR 'fused teeth':ti,ab OR 'fused tooth':ti,ab OR 'supernumerary tooth':ti,ab OR 'supernumerary teeth':ti,ab OR 'anodont*':ti,ab OR 'crowding*':ti,ab OR 'hypodont*':ti,ab OR 'tooth injury':ti,ab OR 'tooth injuries':ti,ab OR 'dental disorder':ti,ab OR 'ectopic teeth':ti,ab OR 'ectopic tooth':ti,ab OR 'impacted molar':ti,ab OR 'impacted tooth':ti,ab OR 'impacted teeth':ti,ab OR 'tooth, impacted':ti,ab OR 'tooth eruption':ti,ab OR 'tooth eruption, ectopic':ti,ab OR 'tooth ankylosis':ti,ab OR 'unerupted tooth'/exp OR 'unerupted tooth' OR 'unerupted tooth':ti,ab OR 'unerupted teeth':ti,ab

'epidemiology'/exp OR 'epidemiolog*':ti,ab OR 'clinical epidemiolog*':ti,ab OR 'epidemiologic factor*':ti,ab OR 'epidemiologic method*':ti,ab OR 'epidemiologic research*':ti,ab OR 'epidemiologic stud*':ti,ab OR 'epidemiologic study design*':ti,ab OR 'epidemiologic characteristic*':ti,ab OR 'epidemiologic survey*':ti,ab OR 'epidemiological research*':ti,ab OR 'epidemiologic model*':ti,ab OR 'epidemiological stud*':ti,ab OR 'epidemiological study design*':ti,ab OR 'incidence'/exp OR 'incidence*':ti,ab OR 'incidence stud*':ti,ab OR 'occurrence*':ti,ab OR 'incidence rate*':ti,ab OR 'prevalence*':ti,ab OR 'prevalence stud*':ti,ab OR 'frequenc*':ti,ab OR 'occurenc*':ti,ab

<u>Search string Web of Science</u>

(Orthodontics OR Orthodont* OR Orthodontia* OR Orthodontic care* OR Orthodontic model* OR Orthodontology*) *AND* (Infant OR Infant* OR Newborn* OR Neonat*: OR Child OR Child* OR Adolescent OR Adolescen* OR Teen* OR Youth OR Minors OR Minor* OR baby* OR high risk infant* OR hospitalized infant* OR newborn* OR preschool child* OR juvenile* OR schoolchild* OR schoolgirl* OR schoolboy* OR school girl* OR school boy* OR teenage* OR infancy* OR childhood* OR pubert*Infant OR Infant* OR Newborn* OR Neonat*: OR Child OR Child* OR Adolescent OR Adolescen* OR Teen* OR Youth OR Minors OR Minor* OR baby* OR high risk infant* OR hospitalized infant* OR newborn* OR preschool child* OR juvenile* OR schoolchild* OR schoolgirl* OR schoolboy* OR school girl* OR school boy* OR teenage* OR infancy* OR childhood* OR pubert*) *AND* (Malocclusion OR Malocclusion* OR Angles Classification* OR Angles classes malocclusion OR dental malocclusion* OR jaw malocclusion* OR jaw occlusion disorder* OR Cross Bite* OR Crossbite OR Forced Bite* OR Tooth Crowding OR Malocclusion, Angle Class I OR Malocclusion, Angle Class I OR Angle Class I OR Malocclusion, Angle Class II OR Angle Class II OR Angle Class II, Division 1 OR Angle Class II, Division 2 OR Overbite OR Overbite* OR Malocclusion,

Angle Class III OR Angle Class III OR Habsburg Jaw* OR Hapsburg Jaw * OR Prognathism, Mandibular* OR Prognath* OR Underbite* OR Maxillary Discrepanc* OR occlusion disorder, jaw* OR occlusion,mal* OR open bite* OR lower jaw prognath* OR mandible prognath* OR mandible protrusion* OR mandibula prognath* OR mandibular prognath* OR mandibular protrusion* OR progenia* Jaw Abnormalities OR Jaw Abnormalit* OR Cleft Palate OR Cleft lip* OR Cleft Nose OR Micrognath* OR Pierre Robin Syndrome* OR Retrognath* OR Mandibular Retroposition* OR Mandibular Retrusion* OR Maxillary Retroposition* OR Maxillary Retrusion* OR Craniofacial Abnormalities OR Craniofacial Abnormalit* OR Abnormalities, Craniofacial* OR Abnormality, Craniofacial* OR Maxillofacial syndromes OR Maxillofacial syndrome* OR Marfan syndrome OR Marfan syndrome* OR receding jaw* OR Jaw malformation* OR Goldenhar syndrome* OR hemifacial macrosom* OR mandible hypoplas* OR mandibulofacial dysostos* OR maxilla hypoplas* OR micrognath* OR Nager acrofacial dysostos* OR Pierre Robin syndrome* OR chin malformation* OR dysostosis mandibul* OR jaw abnormal* OR jaw anomal* OR jaw dismorfit* OR jaw deformit* OR jaw dismorfit* OR malformation,chin* OR malformation jaw* OR mandible deformit* OR mandible condyle agenes* OR mandibular defect* OR mandibular dysostos* OR mandibular malformat* OR Lip malformation* OR Cleft lip OR Cleft lip* OR Lip, Cleft* OR Lips, Cleft* OR Harelip* OR Cleft Palate OR Cleft Palate* OR Palate Cleft* OR Palates, Cleft* OR cleft lip, face, palate* OR cleft lip, nose* OR cleft lip palate* OR long philtrum* OR unilateral cleft lip* OR bilateral cleft lip* OR Van der Woude syndrome* cleft lip palate* OR cheilo gnathopalatoschis* OR cheilognathopalathoschis* OR cheilognathopalatoschis* OR cheilognatopalatoschiz* OR cheilognathouranoschis* OR cheilognatopalatoschis* OR cheilopalatoschis* OR cleft lip maxillo palate* OR harelip* OR lip jaw palate cleft* OR cleft lip face palate* OR cleft lip nose* OR cleft lip face palate* OR cleft palate lift* OR labiopalatoschis* OR palatolabioschis* OR unilateral cleft lip* OR cleft lip, unilateral* OR lip, unilateral cleft* OR Palate malformation* OR palate malformation* OR cleft lip face palate* OR cleft palate* OR high arched palate* OR Roberts syndrome* OR velocardiofacial syndrome OR CLP OR CP OR UCLP OR Tooth Abnormalities OR Tooth Abnormalit* OR Teeth Abnormalit* OR tooth disease* Tooth Malformation* OR acro-dermato-ugual-lacrimal-tooth syndrome* OR amalogenesis imperfecta* OR dens evaginatus* OR enamel hypoplasia* OR invaginated tooth* invaginated tooth*OR oculodentodigital syndrome* OR Odontome* OR Tooth Supernumerary* OR Tooth Crowding* OR Anodontia* OR Agenes* OR Hypodontia* OR Oligodontia* OR Dens in dent* OR Dental Enamel Hypoplasia* OR Molar Incisor Hypomineralization* OR Amelogenesis Imperfecta* OR Congenital Enamel Hypoplas* OR Dentin Dysplasia* OR Hypoplastic Enamel* OR Hypoplasia Dental Enamel* OR Enamel Agenes* OR Enamel Hypoplas* OR Dentinogenesis Imperfecta* OR Diastema* OR Fused Teeth* OR Double Tooth* OR Fused Mandibular Incisor* OR Tooth, Supernumerary* OR Fourth Molar* OR Odontodysplasia* OR Ghost Teeth* OR Ghost Tooth* OR Odontogenic Dypslasia* OR Odontogenesis Imperfecta* OR Odontoma OR Odontom* OR Tooth Ankylosis OR Tooth Ankylos* OR Dentoalveolar Ankylos* OR Dental Ankylos* OR Teeth Ankylos* OR Tooth Eruption OR Tooth Eruption* OR Teething* OR Failure of Tooth Eruption, Primary* OR Posterior Openbite, Familial* OR Primary Retention of Teeth* OR Unerupted Second Primary Molar* OR Primary Failure of Eruption, Nonsyndromic* OR Dental Noneruption* OR Tooth Eruption, Ectopic OR Tooth Eruption, Ectopic* OR Ectopic Tooth Eruption* OR Eruption, Ectopic Tooth* OR Eruptions, Ectopic Tooth* OR Tooth Eruptions, Ectopic* OR Tooth,Impacted OR Tooth, Impacted* OR Impacted Tooth* OR Teeth, Impacted* OR Impacted Teeth* OR Tooth, Unerupted OR Tooth, Unerupted* OR Unerupted Tooth* OR Teeth, Unerupted* OR Unerupted Teeth* OR Mesial Movement of Teeth OR Mesial Movement of Teeth* OR Teeth Mesial Movement* OR Mesial Migration of Teeth* OR Teeth Mesial Migration* OR Mesial Drift of Teeth* OR Teeth Mesial Drift* OR Tooth Abrasion OR Tooth Abrasion* OR Abrasion, Tooth* OR Abrasion, Dental* OR Dental Abrasion* OR Tooth Erosion OR Tooth Erosion* OR Erosion, Tooth* OR Erosions, Tooth OR Tooth Extraction OR Tooth extraction* OR Extraction, Tooth* OR Extractions, Tooth* OR Premature extraction deciduous tooth* OR Premature extraction deciduous teeth* OR Premature extraction primary tooth* OR Primary extraction primary teeth* OR Serial extraction OR Serial extraction* OR Extraction, Serial* OR Extractions, Serial* OR Tooth Fractures OR Tooth Fracture* OR Fracture, Tooth* OR Fractures, Tooth* OR Cracked tooth syndrome OR Cracked tooth syndrome* OR Syndrome, Cracked Tooth* OR Syndromes, Cracked Tooth* OR Tooth Germ OR

Tooth Germ* OR Germ, Tooth* OR Germs, Tooth* OR Tooth Avulsion OR Tooth Avulsion* OR Avulsion, Tooth* OR Avulsions, Tooth* OR Avulsed Tooth* OR Tooth, Avulsed* OR Dislocation, Tooth* OR Dislocations, Tooth* OR Tooth Dislocation* OR Tooth Luxation* OR Luxation, Tooth* OR Luxations, Tooth* OR Transposition tooth* OR Transposition teeth* OR Tooth, Transposition* OR Teeth, Transposition* OR Tooth Loss OR Tooth Loss* OR Loss, Tooth* OR Premature loss tooth* OR Premature loss deciduous tooth* OR Premature loss teeth* OR Premature loss deciduous teeth* OR Premature loss primary tooth* OR Premature loss primary teeth* OR Tooth Migration OR Migration, Tooth* OR Tooth Drifting* OR Tooth Drift* OR Tooth Mobility OR Tooth Mobilit* OR Mobilities, Tooth* OR Mobility, Tooth* OR Tooth Replantation OR Tooth Replantation* OR Replantation, Tooth* OR Replantations, Tooth* OR Reimplantation, Tooth* OR Reimplantations, Tooth* OR Tooth Reimplantation* OR Tooth Resorption OR Tooth Resorption* OR Resorption, Tooth* OR Resorptions, Tooth* OR Tooth Injuries OR Tooth Injur* OR Injuries, Teeth* OR Injury, Teeth* OR Teeth Injur* OR Injuries, Tooth* OR Injury, Tooth* OR Tooth Demineralization OR Tooth Demineralization* OR Tooth Hypomineralization* OR Hypomineralization, Tooth* OR Hypomineralizations, Tooth* OR Demineralization, Tooth* OR Dental Enamel Hypoplasia OR Dental Enamel Hypoplasia* OR Hypoplastic Enamel* OR Enamel, Hypoplastic* OR Enamel Hypoplasia, Dental* OR Hypoplasia, Dental Enamel* OR Enamel Agenesis* OR Ageneses, Enamel* OR Agenesis, Enamel* OR Enamel Ageneses* OR Enamel Hypoplas* OR Hypoplasia, Enamel* OR Hypoplasias, Enamel* OR Molar Incisor Hypomineralization* OR Hypomineralization, Molar Incisor* OR Tooth Attrition OR Tooth Attrition* OR Attrition, Tooth* OR Dental Attrition* OR Occlusal Wear* OR Wear, Occlusal* OR Wears, Occlusal* OR Attrition, Dental* OR Dental Caries OR Dental Caries* OR Dental Decay* OR Caries, Dental* OR Decay, Dental* OR Carious Dentin* OR Dentin, Carious* OR Dentins, Carious* OR Dental White Spot* OR White Spots, Dental* OR White Spot* OR Spot, White* OR Spots, White* OR Dental White Spots* OR White Spot, Dental* OR dental abnomal* OR tooth anomal* OR tooth deformit* OR tooth dysplas* OR fused teeth* OR fused tooth* OR supernumerary tooth* OR supernumerary teeth*OR anodontia* OR crowding* OR dental caries* OR hypodont* OR tooth injur* OR dental disorder* OR ectopic teeth* OR ectopic tooth* OR impacted molar* OR impacted tooth* OR impacted teeth* OR tooth, impacted* OR tooth eruption* OR tooth eruption, ectopic* OR tooth luxation* OR tooth resorption* OR tooth root resorption* OR tooth wear* OR tooth ankylose* OR premature tooth loss* OR premature loss deciduous tooth* OR premature loss deciduous teeth* OR unerupted tooth* OR unerupted teeth*) *AND* (Epidemiology OR Epidemiolog* OR Epidemiologic Studies OR Epidemiologic Stud* OR Epidemiological Stud* OR epidemiological stud* OR epidemiological study design* OR Epidemiologic research design OR Epidemiologic research design* OR Epidemiologic Methods OR Epidemiologic Method* OR Epidemiol* OR clinical epidemiolog* OR cohort effect OR confounding factor* OR epidemiologic* OR epidemiologic factor* OR epidemiologic method* OR epidemiologic research* OR epidemiologic study design* OR epidemiologic characteristic* OR epidemiologic survey* OR epidemiological research* OR epidemiological research design* OR epidemiologic model* OR epidemiometr* OR Incidence OR Incidence* OR Incidence studies OR Incidence stud* OR incidence rate* OR Occurrence* OR Prevalence OR Prevalence* OR Prevalence studies OR Prevalence stud* OR prevalence* OR Frequenc* OR Occurenc*)

<u>Search string Open Grey</u>

Orthodontics OR Orthodont* OR orthodontia* OR orthodontic care*OR orthodontic model* OR orthodontology*

Infant* OR Newborn* OR Neonat* OR Child* OR Adolescent OR Adolescen* OR Teen* OR Youth OR Minor* OR baby* OR high risk infant* OR hospitalized infant* OR newborn* OR preschool child* OR juvenile* OR schoolchild* OR schoolgirl* OR schoolboy* OR school girl* OR school boy* OR teenage* OR infancy* OR childhood* OR pubert*

Malocclusion* OR Angle’s Classification* OR Cross Bite* OR Crossbite* OR Forced bite* OR Tooth Crowding* OR Maxillary Discrepancy* OR Angle’s Classification*OR Angle’s classes malocclusion* OR Malocclusion, Angle Class I*OR Angle Class I* OR Malocclusion, Angle Class II* OR Angle Class

II* OR Angle Class II, Division 1* OR Angle Class II, Division 2*OR Class II Malocclusion, Division 1* OR Class II Malocclusion, Division 2* OR Malocclusion, Angle Class II, Division 1 *OR Malocclusion, Angle Class II, Division 2* OR Overbite*OR Malocclusion, Angle Class* OR Angle Class III* OR Habsburg Jaw* OR Hapsburg Jaw* OR Prognathism, Mandibular* OR Mandibular Prognathism*OR Prognathism* OR Underbite* OR Jaw Abnormalit* OR Jaw Abnormal* OR Cleft Palate* OR Cleft Lip and Palate* OR Cleft lip,Palate* OR Cleft Lip and Nose and Palate* OR Cleft Lip, Nose, Palate* OR Cleft Lip, Nose and Palate* OR Micrognathism * OR Pierre Robin Syndrome* OR Prognathism* OR Retrognathia* OR Mandibular Retroposition* OR Mandibular Retrusion* OR Maxillary Retroposition* OR Maxillary Retrusion* OR Retrognathism* OR Tooth Abnormalit* OR Teeth Abnormalit* OR Anodontia* OR Dens in Dente* OR Dental Enamel Hypoplasia* OR Amelogenesis Imperfecta* OR Dentin Dysplasia* OR Dentinogenesis Imperfecta* OR Diastema* OR Fused Teeth* OR Tooth, Supernumerary* OR Odontodysplas* OR Tooth supernumerary* OR Odontom* OR Tooth Crowding* OR Tooth Ankylosis* OR Ankylosis of Teeth* OR Ankylosis, Dentoalveolar* OR Ankylosis, Tooth* OR Dental Ankylosis* OR Dentoalveolar Ankylosis* OR Tooth Eruption* OR Tooth Eruption,Ectopic* OR Tooth, Impacted*OR Teeth, Impacted* OR Tooth, Unerupted* OR Teeth, unerupted * OR Mesial Movement of Teeth* OR Tooth Abrasion* OR Tooth Discoloration* OR Tooth Erosion* OR Tooth Extraction* OR Premature extraction deciduous teeth and - tooth and - primary teeth and – tooth* OR Tooth Fractures* OR Tooth Germ* OR Tooth Avulsion*OR Avulsed Tooth*OR Dislocation, Tooth*OR Tooth Luxation* OR Tooth Loss* OR Tooth, premature loss* OR Tooth Migration* OR Tooth Drift* OR Tooth Mobility* OR Tooth Replantation* OR Tooth Resorption* OR Tooth Injuries* OR Injuries, Teeth* OR Injuries, Tooth* OR Teeth Injuries* OR Tooth, Deciduous* OR Tooth, Permanent* OR Tooth Demineralization* OR Dental Enamel Hypoplasia* OR Tooth Attrition* OR Dental Caries* OR Tooth Caries* OR Carious Dentin* OR Dental Decay* OR Dental White Spot* OR Dental White Spots* OR White Spots* OR Syndrome* OR Congenital Abnormalities* OR Congenital Abnormality* OR Birth Defects* OR Congenital Defects* OR Congenital Defect* OR Defomit* OR Congenital Disorder* OR OR Maxillofacial Syndrom* OR Marfan syndrome* OR Craniofacial Abnormal* OR Jaw malformation* OR Goldenhar syndrome* OR hemifacial microsomia* OR mandibular hypoplasia* OR mandibulofacial dysostosis* OR maxillar hypoplasia* OR micrognathia* OR Nager acrofacial dysostosis* OR Pierre Robin syndrome* OR Lip malformation* OR Cleft lip* OR Cleft Palate* OR Cleft Lip, Face, Palate* OR Cleft Lip Nose* OR Cleft Lip, Palate* OR Long Philtrum* OR Unilateral Cleft Lip* OR Van der Woude syndrome* OR Palate malformation* OR Cleft lip, face, palate* OR cleft palate* OR High arched palate* OR Roberts syndrome* OR Van de Woude syndrome* OR Velocardiofacial syndrome* OR Tooth malformation* OR acro-dermato-ugual-lacrimal-tooth syndrome* OR amalogenesis imperfecta* OR dens evaginatus* OR enamel hypoplasia* OR invaginated tooth* OR oculodentodigital syndrome* OR oligodontia* OR Van der Woude syndrome*

Epidemiologic Studies* OR Epidemiological Studies* OR Epidemiolog* Stud* OR Epidemiologic research design* OR Epidemiologic Research Designs* OR Epidemiological Research Design* OR Epidemiologic Methods* OR DMF Ind* OR Indexe, DMF* OR Epidemiology* OR Epidemiol* OR Frequenc* OR Incidence* OR Incidence studies* OR Occurrenc* OR Prevalence* OR Prevalence studies* OR Frequenc* OR Occurrenc*

Additional file S12: PRISMA 2020 Checklist

Section and Topic	Item #	Checklist item	Location where item is reported
**TITLE**			
Title	1	Prevalence of Orthodontic Malocclusions in Healthy Children and Adolescents: A Systematic Review, Page 1 (Tittle)	
**ABSTRACT**			
Abstract	2	Page 1 (Abstract)	
**INTRODUCTION**			
Rationale	3	Page 1, 2	
Objectives	4	Page 1, 2	
**METHODS**			
Eligibility criteria	5	Page 3 (2.3. Eligibility Criteria)	
Information sources	6	Page 2 (2.2. Search Strategy)	
Search strategy	7	Page 2 (2.2. Search Strategy)	
Selection process	8	Page 3 (2.5. Study Selection)	
Data collection process	9	Page 3 (2.5. Data Collection and Analysis)	
Data items	10a	Page 3 (2.5. Data Collection and Analysis)	
	10b	Page 3 (2.5. Data Collection and Analysis)	
Study risk of bias assessment	11	Page 3 (2.5. Risk of Bias Assessment)	
Effect measures	12	Page 12-22 (3.3. Prevalence of Malocclusion, 3.3.1. Sagittal Occlusion, 3.3.2 Vertical Occlusion, Table 2. Prevalence of overjet, reversed overjet, overbite, and open bite, 3.3.3. Transversal Occlusion, 3.3.4. Tooth Anomalies, 3.3.5. Space anomalies, 3.3.6. Oral Habits, Table 3. Prevalence of oral habits, 3.3.7. Geographic Differences, Table 4. Prevalence of Angle classification and deciduous molar occlusion according to geographical location, Table 5. Prevalence of different transversal malocclusions and anterior crossbite according to geographical location, Table 6. Prevalence of tooth anomalies according to geographical location, Table 7. Prevalence of space anomalies according to geographical location)	
Synthesis methods	13a	Only weighted means and standard deviations were calculated and noted in the tables and results, because of the heterogeneity of the data, the included studies were thought to be too different in terms of methodological approaches (f.e. evaluation of different metrics and outcomes, different participants and settings).<br>Page 12-22 (3.3. Prevalence of Malocclusion, 3.3.1. Sagittal Occlusion, 3.3.2 Vertical Occlusion, Table 2. Prevalence of overjet, reversed overjet, overbite, and open bite, 3.3.3. Transversal Occlusion, 3.3.4. Tooth Anomalies, 3.3.5. Space anomalies, 3.3.6. Oral Habits, Table 3. Prevalence of oral habits, 3.3.7. Geographic Differences, Table 4. Prevalence of Angle classification and deciduous molar occlusion according to geographical location, Table 5. Prevalence of different transversal malocclusions and anterior crossbite according to geographical location, Table	

Section and Topic	Item #	Checklist item	Location where item is reported
		6. Prevalence of tooth anomalies according to geographical location, Table 7. Prevalence of space anomalies according to geographical location)	
	13b	All prevalence were noted in percentages, which were rounded to one digit after the decimal point.	
	13c	The parameters were noted as in the described study, with the prevalence in percentages, rounded to one digit after the decimal point.	
	13d	Only weighted means and standard deviations were calculated and noted in the tables and results, because of the heterogeneity of the data, the included studies were thought to be too different in terms of methodological approaches (f.e.evaluation of different metrics and outcomes, different participants and settings).<br>Page 12-22 (3.3. Prevalence of Malocclusion, 3.3.1. Sagittal Occlusion, 3.3.2 Vertical Occlusion, Table 2. Prevalence of overjet, reversed overjet, overbite, and open bite, 3.3.3. Transversal Occlusion, 3.3.4. Tooth Anomalies, 3.3.5. Space anomalies, 3.3.6. Oral Habits, Table 3. Prevalence of oral habits, 3.3.7. Geographic Differences, Table 4. Prevalence of Angle classification and deciduous molar occlusion according to geographical location, Table 5. Prevalence of different transversal malocclusions and anterior crossbite according to geographical location, Table 6. Prevalence of tooth anomalies according to geographical location, Table 7. Prevalence of space anomalies according to geographical location)	
	13e	No subgroup analysis nor meta-analysis were performed to explore possible causes of heterogeneity among study results because the heterogeneity of the methods and study samples used in the different included studies.	
	13f	No sensitivity analyses was conducted to assess robustness of the synthesized results.	
Reporting bias assessment	14	Page 3 (2.5. Risk of Bias Assessment)).	
Certainty assessment	15	Page 3 (2.5. Risk of Bias Assessment)	
**RESULTS**			
Study selection	16a	Page 4 (Fig 1. PRISMA flow diagram)	
	16b	Cite studies that might appear to meet the inclusion criteria, but which were excluded, and explain why they were excluded.	
Study characteristics	17	Page 4, 6-10 (3.1. Characteristics of the Studied Population, Table 1. Characteristics of the included studies)	
Risk of bias in studies	18	Page 22-26 (3.4. Risk of Bias, Table 8 Risk of BIAS assessment according to the MINORS tool, Fig. 2. Risk of Bias Assessment for Non-Comparative studies, Fig 3. Risk of Bias Assessment for Comparative studies)	
Results of individual studies	19	Page 12-22 (3.3. Prevalence of Malocclusion, 3.3.1. Sagittal Occlusion, 3.3.2 Vertical Occlusion, Table 2. Prevalence of overjet, reversed overjet, overbite, and open bite, 3.3.3. Transversal Occlusion, 3.3.4. Tooth Anomalies, 3.3.5. Space anomalies, 3.3.6. Oral Habits, Table 3. Prevalence of oral habits, 3.3.7. Geographic Differences, Table 4. Prevalence of Angle classification and deciduous molar occlusion according to geographical location, Table 5. Prevalence of different transversal malocclusions and anterior crossbite according to geographical location, Table 6. Prevalence of tooth anomalies according to geographical location, Table 7. Prevalence of space anomalies according to geographical location)	

Section and Topic	Item #	Checklist item	Location where item is reported
Results of syntheses	20a	Page 22-26 (3.4. Risk of Bias, Table 8 Risk of BIAS assessment according to the MINORS tool, Fig. 2. Risk of Bias Assessment for Non-Comparative studies, Fig 3. Risk of Bias Assessment for Comparative studies)	
	20b	Only weighted means and standard deviations were calculated and noted in the tables and results, because of the heterogeneity of the data, the included studies were thought to be too different in terms of methodological approaches (f.e. evaluation of different metrics and outcomes, different participants and settings).<br>Page 12-22 (3.3. Prevalence of Malocclusion, 3.3.1. Sagittal Occlusion, 3.3.2 Vertical Occlusion, Table 2. Prevalence of overjet, reversed overjet, overbite, and open bite, 3.3.3. Transversal Occlusion, 3.3.4. Tooth Anomalies, 3.3.5. Space anomalies, 3.3.6. Oral Habits, Table 3. Prevalence of oral habits, 3.3.7. Geographic Differences, Table 4. Prevalence of Angle classification and deciduous molar occlusion according to geographical location, Table 5. Prevalence of different transversal malocclusions and anterior crossbite according to geographical location, Table 6. Prevalence of tooth anomalies according to geographical location, Table 7. Prevalence of space anomalies according to geographical location)	
	20c	The heterogeneity of the data, the included studies were too different in terms of methodological approaches (f.e. evaluation of different metrics and outcomes, different participants and settings), this results in bias in the used methodology, especially the different definitions and values for different measurements of orthodontic parameters and in selection bias, f.e. participants (pre-)schoolchildren versus patients.	
	20d	Not performed	
Reporting biases	21	Selection bias: The use of patient samples can also introduce additional bias over random samples since patients seek dental or orthodontic treatment for a reason. In this sense, it is preferable to conduct an epidemiological study on a population-based sample rather than on patient populations.<br>Reporting bias: A large variety In methods used to assess the different orthodontic features, resulted in a heterogeneity in the reporting of the same parameters	
Certainty of evidence	22	Page 23, 3.4. Risk of Bias<br>The risk of bias of the included articles determined according to the MINORS tool is shown in Table 8. The scores of each article are plotted in Figures 2 and 3 for non-comparative and comparative studies, respectively, and are sorted by publication year, from oldest to newest. The lowest score for non-comparative studies was 2, and the highest was 10, with a possible maximum score of 16. For comparative studies, the lowest score was 5, and the highest was 13, with a possible maximum of 24. A very discrete tendency to better article quality over time can be found in both comparative and non-comparative studies.	
**DISCUSSION**			
Discussion	23a	Page 26-28 (4. Discussion)	
	23b	Page 26-26 (4. Discussion)	
	23c	Page 26-28 (4. Discussion)	
	23d	Page 26-28 (4. Discussion, 5. Conclusions)	
**OTHER INFORMATION**			
Registration and	24a	The protocol was registered in the international prospective register of systematic reviews (PROSPERO) under protocol registration number CRD42018086464.	

Section and Topic	Item #	Checklist item	Location where item is reported
protocol	24b	Additional file 10.	
	24c	Not applicable	
Support	25	No funding received	
Competing interests	26	Lutgart De Ridder declares that she has no conflict of interest. Antonia Aleksieva declares she has no conflict of interest. Guy Willems declares that he has no conflict of interest. Dominique Declerck declares that she has no conflict of interest. Maria Cadenas de Llano Perula declares that she has no conflict of interest.	
Availability of data, code and other materials	27	Template data collection forms: not available; data extracted from included studies: additional table 2,3,4,5,6,7,8,9 data used for all analyses: not available, analytic code: not available, any other materials used in the review: Excluded studies and reason for exclusion: additional table 1.	

From: Page MJ, McKenzie JE, Bossuyt PM, Boutron I, Hoffmann TC, Mulrow CD, et al. The PRISMA 2020 statement: an updated guideline for reporting systematic reviews. BMJ 2021;372:n71. doi: 10.1136/bmj.n71

For more information, visit: http://www.prisma-statement.org/
